# Ligand-receptor promiscuity enables cellular addressing

**DOI:** 10.1016/j.cels.2022.03.001

**Published:** 2022-05-18

**Authors:** Christina J. Su, Arvind Murugan, James M. Linton, Akshay Yeluri, Justin Bois, Heidi Klumpe, Matthew A. Langley, Yaron E. Antebi, Michael B. Elowitz

**Affiliations:** 1 Division of Biology and Biological Engineering, California Institute of Technology, Pasadena, CA 91125, USA; 2 Department of Physics, University of Chicago, Chicago, IL 60637, USA; 3 Division of Chemistry and Chemical Engineering, California Institute of Technology, Pasadena, CA 91125, USA; 4 Department of Molecular Genetics, Weizmann Institute of Science, Rehovot 76100, Israel; 5 Department of Applied Physics, California Institute of Technology, Pasadena, CA 91125, USA; 6 Howard Hughes Medical Institute, Chevy Chase, MD 20815, USA

**Keywords:** bone morphogenetic protein, BMP, signaling pathways, promiscuity, combinatorial signaling, ligand-receptor interactions, communication systems, signal processing, information theory, cell-type specificity

## Abstract

In multicellular organisms, secreted ligands selectively activate, or “address,” specific target cell populations to control cell fate decision-making and other processes. Key cell-cell communication pathways use multiple promiscuously interacting ligands and receptors, provoking the question of how addressing specificity can emerge from molecular promiscuity. To investigate this issue, we developed a general mathematical modeling framework based on the bone morphogenetic protein (BMP) pathway architecture. We find that promiscuously interacting ligand-receptor systems allow a small number of ligands, acting in combinations, to address a larger number of individual cell types, defined by their receptor expression profiles. Promiscuous systems outperform seemingly more specific one-to-one signaling architectures in addressing capability. Combinatorial addressing extends to groups of cell types, is robust to receptor expression noise, grows more powerful with increases in the number of receptor variants, and is maximized by specific biochemical parameter relationships. Together, these results identify design principles governing cellular addressing by ligand combinations.

## Highlights

• Ligand combinations can selectively activate cell types with varying receptor profiles
• Models show how such “addressing” arises from promiscuous ligand-receptor interactions
• Ligand combinations can also address complex combinations of cell types
• Anticorrelated biochemical parameter regimes enhance the addressing capacity

## Graphical abstract

**Figure:**
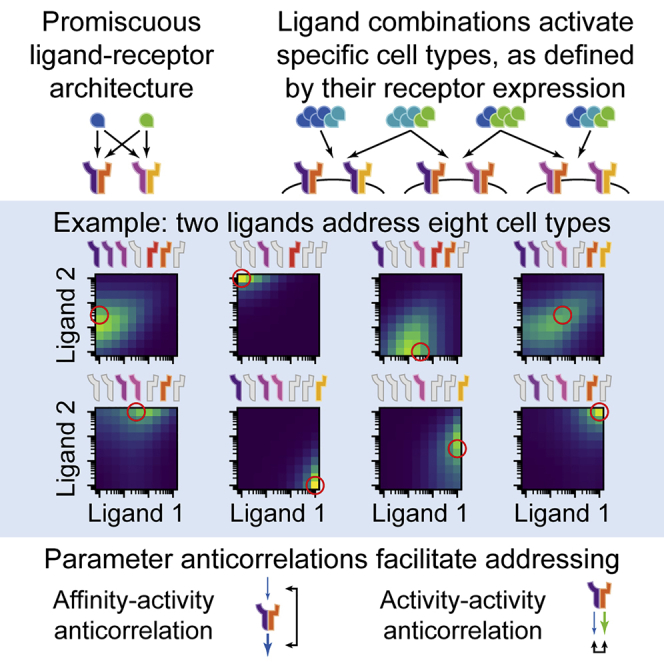

## Teaser

Many signaling pathways comprise sets of ligand and receptor variants that interact promiscuously. Mathematical modeling and experiments with the BMP pathway reveal how these interactions allow ligand combinations to selectively activate, or “address,” individual cell types or groups of cell types based on their combinatorial receptor expression profiles.

## Introduction

During development, a handful of core communication pathways control a huge range of cell fate decisions and other processes across diverse tissues and contexts. These pathways include the bone morphogenetic protein (BMP) and the broader transforming growth factor β (TGF-β) pathways, as well as Wnt, fibroblast growth factor (FGF), Hedgehog, and Notch. Each of these pathways comprises multiple ligand and receptor variants that are expressed in different combinations in different cell types. The expression of these pathway components is generally widespread, with the receptors for most pathways being expressed in most cell types and the ligands for most pathways being present in most tissues. Puzzlingly, despite the ubiquitous expression of their signaling components, the activation of pathways tends to be tightly restricted, occurring only in specific cell types within particular spatiotemporal contexts. If we understood the principles that naturally restrict signaling to specific cell types, we could potentially apply them to control pathways with greater cell-type specificity in therapeutic applications.

Multiple mechanisms have been shown to restrict pathway activation. First, the modulation of extracellular ligand concentrations through the formation of morphogenetic gradients, secreted inhibitors, and factors in the extracellular matrix allows spatial and temporal control of signaling (Bier and De Robertis, 2015; Rogers and Schier, 2011). Second, intracellularly, cells can regulate the amplitude and dynamics of their individual pathway responses by controlling the phosphorylation of effector proteins, subcellular localization of signaling components, or selective silencing of particular target gene sets (Axelrod et al., 1998; Lim-Tio and Fuller, 1998; Shaul and Seger, 2007). Third, different ligand variants could bind to and interact with different receptor variants with different strengths. This mechanism would allow the organism to use different ligands to preferentially activate different cell types based on the receptor variants they express. These three mechanisms could operate individually or in combination.

Here, we focus on this third class of mechanism. It is well known that different ligands can preferentially bind to and activate different receptors and that different ligands can activate different downstream target genes in the same cell type (Nandagopal et al., 2018; Wootten et al., 2018). However, the features that determine the number of distinct cell types or cell-type combinations that can be selectively activated using a given set of ligands are not understood. This level of specificity describes the encoding of information about which cell types, among the constellation of cell types in a complex tissue or entire body, will activate in response to the ligand-encoded “message.” Therefore, we introduced the term “addressing” to denote the ability of ligands to selectively activate, or “address,” a pathway in different cell types or cell-type combinations.

**Figure 1:**
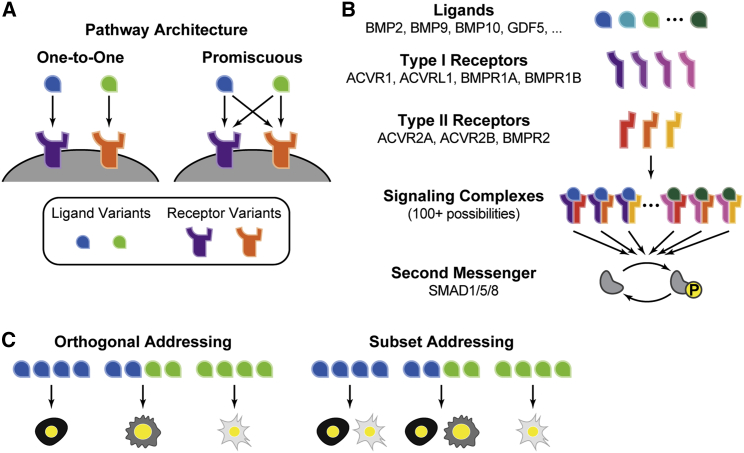
Promiscuous ligand-receptor interactions in the BMP pathway may allow combinatorial addressing (A) In a one-to-one ligand-receptor architecture (left), each ligand interacts exclusively with a single receptor, whereas in a promiscuous architecture (right), ligands interact with multiple receptor variants. (B) In this simplified schematic of the BMP pathway, ligands interact combinatorially with type I and type II receptors at the cell membrane to form signaling complexes, which then activate SMAD1/5/8 effector proteins. (C) Signaling pathways could enable different forms of addressing. In orthogonal addressing (left), different combinations of ligands each activate a distinct cell type. More generally, subset addressing (right) could allow the activation of different groups of cell types by different ligand combinations.

The simplest conceivable implementation of addressing uses specific, one-to-one ligand-receptor interactions, where each ligand variant interacts exclusively with a single cognate receptor variant (Figure 1A, left). This architecture is conceptually straightforward, has been implemented synthetically in the synNotch system (Morsut et al., 2016), and is extendable, as new orthogonal ligand-receptor pairs can provide additional communication channels without disrupting the existing ones. However, most of the natural signaling pathways do not exhibit one-to-one ligand-receptor interactions. Instead, they employ a many-to-many, or promiscuous, architecture in which each ligand variant interacts with multiple receptor variants and vice versa (Figure 1A, right). Pathways such as BMP (Heldin et al., 1997; Massagué, 1998; Mueller and Nickel, 2012; Nickel and Mueller, 2019; Schmierer and Hill, 2007), Wnt (Llimargas and Lawrence, 2001; Wodarz and Nusse, 1998), Notch (Shimizu et al., 2000a, 2000b), Eph-Ephrin (Dai et al., 2014), and FGF (Ornitz et al., 1996; Zhang et al., 2006) all exhibit promiscuous interactions among their multiple ligand and receptor variants. It has generally remained unclear whether molecular promiscuity in ligand-receptor interactions is compatible with addressing at all and, if so, whether it might counterintuitively provide potential advantages compared with simpler one-to-one architectures. More generally, a concise set of principles governing the design of multi-ligand, multi-receptor interaction systems has not been identified.

The BMP pathway provides an ideal system to study these questions. BMP plays diverse roles in most tissues and has demonstrated therapeutic potential (David and Massagué, 2018; Massagué, 2000; Miyazono et al., 2010; Wagner et al., 2010; Wang et al., 2014). In well-studied systems, individual cells co-express multiple receptor variants and are simultaneously exposed to multiple ligand variants, suggesting that the pathway could function combinatorially (Diez-Roux et al., 2011; Dudley and Robertson, 1997; Godin et al., 1999; Graham et al., 2014; Kapushesky et al., 2010; Li and Ge, 2011; Liem et al., 1995; Simic and Vukicevic, 2005; Zhang et al., 1998). These ligand and receptor variants have been shown to interact promiscuously. In mammals, the pathway comprises more than ten distinct homodimeric and heterodimeric ligand variants as well as four type I and three type II receptor variants (Massagué, 2000; Miyazono et al., 2010; Shi and Massagué, 2003). Signaling complexes, comprising a covalent ligand dimer and two type I and two type II receptor subunits, phosphorylate SMAD1/5/8 effectors, which translocate to the nucleus and act as transcription factors to control the expression of target genes (Figure 1B). Overall, this pathway architecture uses combinations of receptors to integrate information from combinations of ligands.

Previous observations suggest that the BMP system can generate complex ligand- and cell-type-dependent pathway activation patterns (Baur et al., 2000; Chen et al., 2013; Grassinger et al., 2007; Lind et al., 1996; Varley and Maxwell, 1996; Yu et al., 2008). For example, during neural tube development, different BMP ligands, expressed in overlapping combinations, direct distinct dorsal interneuron identities in neural progenitors, with each ligand showing specific effects on a subset of interneuron identities but not others (Andrews et al., 2017). This behavior could result from the addressing of different progenitor states by distinct ligand combinations and/or by ligand-specific activation of different target programs.

Recently, mathematical modeling, together with *in vitro* experiments, showed that the competitive formation of distinct BMP signaling complexes with different ligands and receptors effectively generates a set of “computations” in which pathway activity depends on the relative concentrations and identities of multiple ligands (Antebi et al., 2017; Klumpe et al., 2022; Martinez-Hackert et al., 2021). These computations comprise distinct response functions, including ratiometric and additive responses as well as imbalance and balance detection responses that are minimal or maximal, respectively, at defined ligand ratios (Figure S1). Further, the pathway can perform different computations on the same ligands depending on the combinations of receptors expressed by individual cells. These results suggest that promiscuous ligand-receptor interactions might allow addressing to function combinatorially, with different ligand combinations addressing particular cell types based on their receptor expression profiles.

Here, we aim to understand how molecular promiscuity in ligand-receptor interactions could potentially enable the addressing of specific cell types based on their receptor expression profiles. To this end, we developed a minimal mathematical model of promiscuous ligand-receptor interactions. Although additional biochemical mechanisms could further augment the addressing in natural biological systems, focusing on ligand-receptor interactions allowed us to explore and understand the specific capabilities that are introduced by this aspect of the pathway. Using this model, we found that promiscuous ligand-receptor interactions alone are sufficient to generate an extensive repertoire of orthogonal communication channels (Figure 1C, left), with higher specificity than that of the same number of ligands in the simpler one-to-one architecture. Modest increases in the number of receptor variants increase the number and orthogonality of these addressing channels. Furthermore, the promiscuous architecture allows ligand combinations to address not only individual cell types but also more complex groups of cell types (Figure 1C, right). Experimentally, similar types of addressing can be observed in cell lines with differing receptor expression profiles. Finally, using an information theoretic framework, we show how biochemical features, such as anticorrelations between affinity and activity parameters, maximize the information content that can be transmitted through promiscuous ligand-receptor interactions. These results highlight a potentially general biological design principle—promiscuous ligand-receptor interactions enable ligand combinations to address cell types based on the receptor combinations they express—that should be useful for understanding and designing natural and synthetic communication systems.

## Results

### A minimal model allows for the analysis of promiscuous BMP ligand-receptor interactions

**Figure 2:**
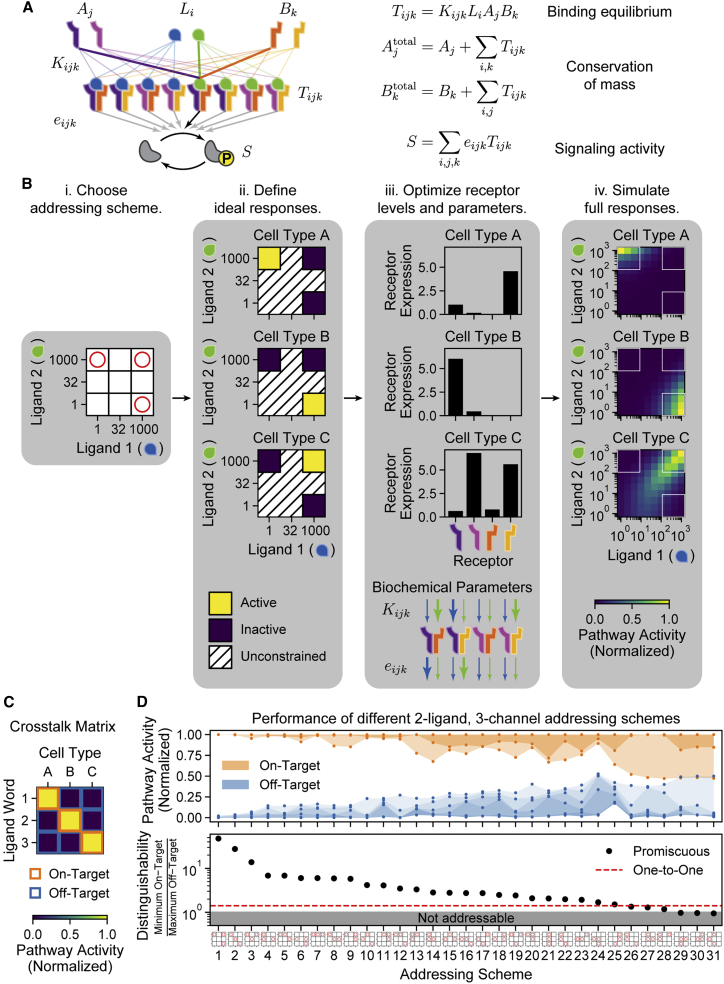
A mathematical model of promiscuous ligand-receptor interactions allows systematic optimization of addressing capabilities (A) A minimal model of the BMP signaling pathway includes ligand variants ($L_{i}$, blue and green), which interact with type I receptors ($A_{j}$, purple and pink) and type II receptors ($B_{k}$, orange and yellow) to form a combinatorial set of trimeric signaling complexes ($T_{ijk}$) with varying affinities ($K_{ijk}$). Active signaling complexes phosphorylate the SMAD effector with varying efficiencies ($e_{ijk}$). Equations describe the steady-state levels of each component and the total signal S (STAR Methods: One-step model for promiscuous interactions). (B) Optimization systematically identifies potential combinatorial addressing schemes in four steps. (i) An orthogonal addressing scheme is specified as orthogonal activation by a set of desired ligand words (red circles). Discretization of ligand space (3 × 3 grid) enables the enumeration of all such addressing schemes. (ii) A given orthogonal addressing scheme can be translated into target response functions in which each cell type is activated by exactly one ligand word (yellow) and not by others (blue). Responses to other ligand words (hatched) are unconstrained. (iii) Least-squares optimization identifies a global set of affinity ($K_{ijk}$) and efficiency ($e_{ijk}$) parameters, along with a set of receptor expression levels for each cell type, which yield responses similar to the target functions. Upper and lower arrows represent affinity and activity parameters, respectively, for each receptor dimer complexed with each of the two ligands (blue and green arrows). Thin and thick arrows correspond to low and high values, respectively. (iv) Responses can be simulated at higher resolution for visualization and further analysis. (C) After optimization, the crosstalk matrix represents the responses of each cell type at the selected ligand words (orthogonal channels). For orthogonal addressing, this matrix should ideally be diagonal, with each ligand word activating only its target cell type (orange border) with no off-target activation (blue border). (D) Best optimization results are shown for all 31 possible three-channel orthogonal addressing schemes (STAR Methods: Enumeration of orthogonal addressing schemes). (Top) Distributions of on-target (orange) and off-target (blue) activation levels are plotted, representing all elements in the crosstalk matrix. Shaded regions span all activity values. (Bottom) The corresponding distinguishability value for each addressing scheme is shown (black). Distinguishability values below 1 (gray region) indicate that the corresponding scheme cannot be successfully addressed. For comparison, the best distinguishability achieved in a one-to-one architecture is shown (red). Addressing schemes (x axis) are shown in order of decreasing distinguishability. See also Figures S1 and S2.

To explore the addressing capacity of promiscuous ligand-receptor systems, we developed a minimal mathematical model based on the architecture of the BMP pathway (STAR Methods: One-step model for promiscuous interactions). Briefly, the model describes a set of $n_{L}$ ligands, $n_{A}$ type I receptors, and $n_{B}$ type II receptors. A ligand $L_{i}$ binds simultaneously to type I and type II receptor subunits $A_{j}$ and $B_{k}$ to form an active signaling complex $T_{ijk}$ (Figure 2A, left). A set of effective interaction strengths, denoted by $K_{ijk}$, represents the strength of the binding between a ligand, a type I receptor subunit, and a type II receptor subunit. We further assume that each signaling complex has its own specific activity, denoted by $e_{ijk}$, controlling the rate at which it phosphorylates downstream SMAD effector proteins. The overall activity of the pathway is then the sum of the concentrations of the signaling complexes, each weighted by its own activity parameter. We assume steady state in ligand-receptor binding and unbinding, which occur at fast time scales relative to the response to signaling. Under this assumption, the model can be described by one set of equations representing binding and unbinding interactions, a second set of equations representing the conservation of total receptor levels, and an expression for the total pathway activity, S (Figure 2A, right).

To solve the model efficiently, we used Equilibrium Toolkit (EQTK), an optimized Python-based numerical solver for biochemical reaction systems (Bois, 2020; Dirks et al., 2007). For simplicity, the model neglects some specific features of the natural BMP pathway, including the sequential binding of ligands to receptors and the hexameric nature of the full BMP signaling complexes (Massagué, 2000; Shi and Massagué, 2003). These features could enable even greater complexity in pathway behavior beyond that described for this minimal model (STAR Methods: Comparison with alternative models). This model was capable of reproducing different response functions that were previously observed experimentally and in a more complex model (Antebi et al., 2017), including ratiometric, additive, imbalance, and balance behaviors (Figure S1).

### An optimization approach identifies possible addressing schemes

Here, using the model, we sought to identify mixtures of ligands at specific concentrations, or “ligand words,” that preferentially activate, or address, specific “cell types,” which are defined here and throughout the paper as a group of cells sharing a common receptor expression profile. (An overview of addressing terminology is provided in Box 1.) We started by searching for instances of “orthogonal addressing,” where each ligand word exclusively activates a single cell type, providing one communication channel per cell type. Intuitively, increasing the number of variants of ligand ($n_{L}$) and receptors ($n_{A}$ and $n_{B}$) should expand the number N of possible channels by allowing for a greater diversity of ligand words and cell types. However, it remains unclear how the number and quality of channels in a promiscuous architecture compares with that possible in a one-to-one architecture, how the number of addressable channels grows with increasing ligand and receptor multiplicity, and what biochemical properties enable optimal orthogonal addressing.

Box 1 **Addressing terminology**

Combinatorial addressing involves mappings between combinations of ligands and responses of cell types defined by their receptor subunit expression. Here, we define some of the terminology introduced in the paper to describe these relationships.

• **Ligand word**: a set of specific concentration values for each ligand variant in a combination. For example, a concentration of 10 μM for ligand 1 and 100 μM for ligand 2 constitutes a ligand word (10 μM, 100 μM).
• **Cell type**: a set of specific receptor subunit expression levels. For example, a cell expressing receptor subunits 1 and 3 would represent a different cell type than a cell expressing subunits 1, 2, and 4 or a cell expressing more subunit 1 and less subunit 3.
• **Channel**: a set of one or more cell types that can be selectively activated (without activating other cell types) by some ligand word. For example, if ligand word 1 activates cell type A, whereas ligand word 2 activates cell types B and C, then "A" and "BC" constitute distinct channels.
• **Bandwidth**: the number of unique channels in a given system. As an example, suppose that ligand words 1 and 2 both activate only cell type A, while ligand word 3 activates cell type B. This system would have a bandwidth of two channels, as ligand words 1 and 2 yield the same activation profile.
• **Combinatorial addressing** (or simply **addressing**): a mapping between ligand words and the corresponding cell type(s) activated by those words.
• **Orthogonal addressing**: a particular form of combinatorial addressing in which each ligand word activates a single, unique cell type. An example of three-channel orthogonal addressing is shown in Figure 2B.
• **Addressing repertoire**: the combinations of cell types (each combination representing a channel) that can be activated across all possible ligand words for a given set of cell types and biochemical parameters. Examples of addressing repertoires are shown with the Venn diagrams in Figure 4A.

The next two terms define quantitative metrics used in this paper.

**Figure 3:**
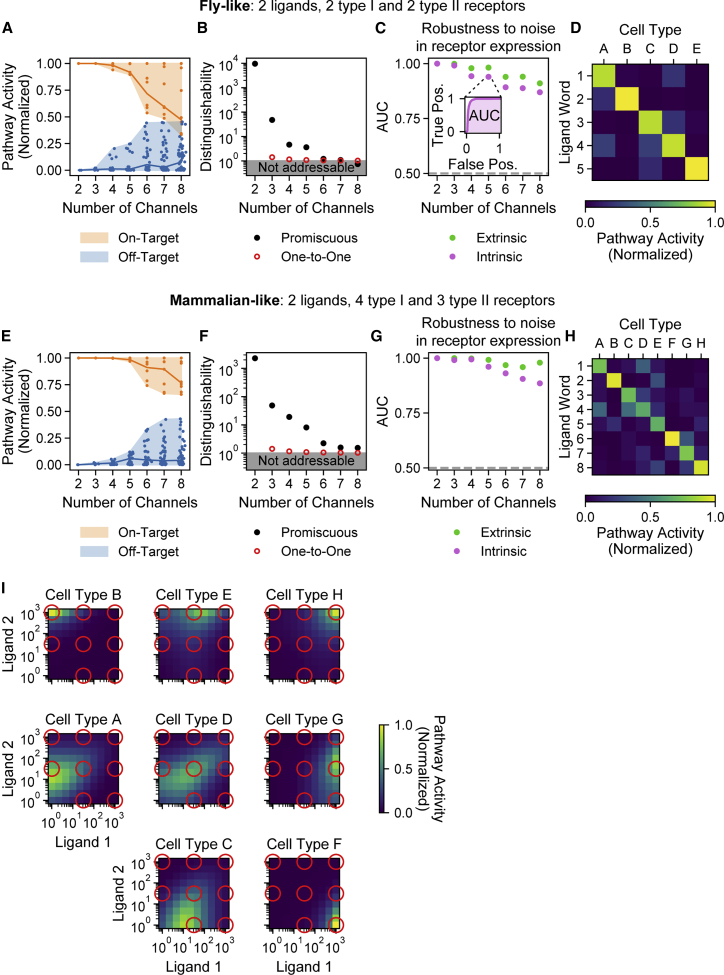
Two ligand variants can independently address eight cell types with high specificity and robustness (A) In the fly-like model with two type I and two type II receptor subunits, the pathway activities of each cell type in response to each ligand word (y axis) are plotted for varying numbers of channels (x axis), using the optimal parameters for each bandwidth. Shaded regions span full distribution of on-target (orange) and off-target (blue) activities, and lines indicate median values. (B) Distinguishability values are plotted for each number of channels (black), together with the optimal values achieved for the same bandwidths in a one-to-one architecture (red). The five-channel system is further analyzed in (D). (C) Robustness to receptor expression fluctuations was evaluated for the top-performing system of each bandwidth. Optimized receptor expression levels were perturbed in a correlated or uncorrelated way to represent, respectively, extrinsic (green) or intrinsic (purple) noise, with a coefficient of variation of 0.5. The resulting receiver operating characteristic (ROC) curves are computed by comparing true and false positive rates for classifying on- and off-target values at different thresholds (inset), and the corresponding area under the curve (AUC) values are plotted for each bandwidth. A perfect classifier has AUC 1, and a random classifier has AUC 0.5 (gray dashed line). (D) The crosstalk matrix shows the response of each cell type at each ligand word of interest for the five-channel example from (A)–(C). Perfect orthogonal specificity would yield a diagonal matrix. (E)The pathway activities for a mammalian-like model with four type I and three type II receptors are shown, as in (A). (F) As in (B), distinguishability values are plotted for the mammalian-like model from (E) (black), along with the optimal values achieved for the same bandwidths in a one-to-one architecture (red). The eight-channel system is further analyzed in (H) and (I). (G) AUC values for the top parameter set of each bandwidth are shown, as in (C). (H) The crosstalk matrix for the eight-channel system in the mammalian-like model is shown, as in (D). (I) The full responses of each cell type are shown for the eight-channel system analyzed in (H). Red circles correspond to the eight ligand words, and cell types are spatially arranged according to the ligand word to which they preferentially respond. For example, the bottom right cell type (cell type F) is orthogonally activated by high levels of ligand 1 only, whereas the top right cell type (cell type H) would be activated by combining high levels of ligand 1 and 2 together. The bottom left ligand word, with low levels of both ligands, is non-activating and, therefore, omitted. See also Figures S3–S5.

**Figure 4:**
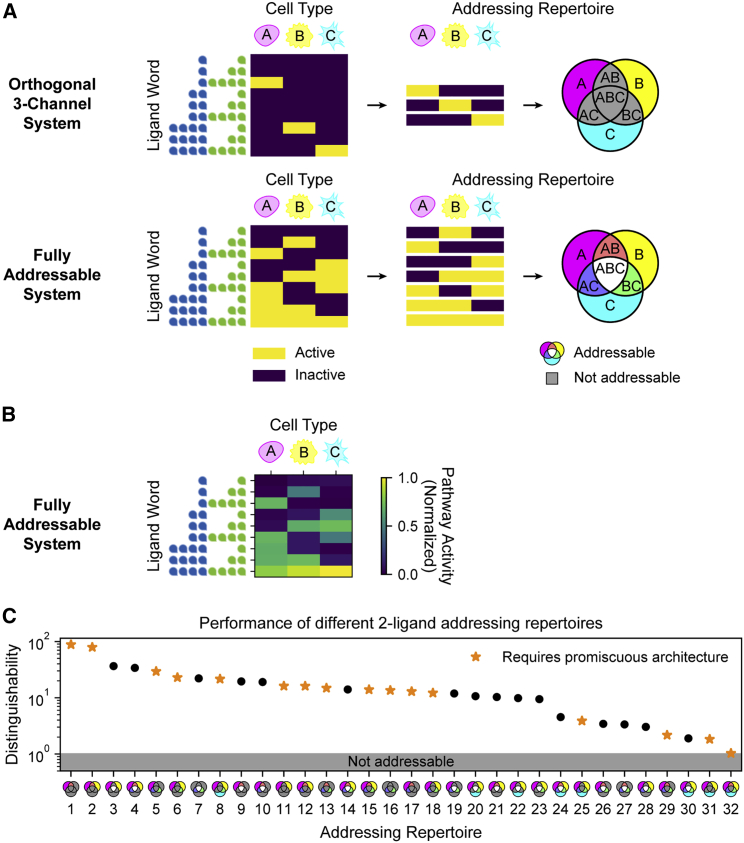
Promiscuous architecture enables diverse addressing repertoires (A) For different parameter sets, the responses of three cell types (A, magenta; B, yellow; and C, cyan) to a titration of two ligands (blue and green) are shown (left). Unique rows reveal the subsets of cell types that can be activated across all ligand words (center). Addressable subsets can also be represented as a Venn diagram (right), where colored regions represent subsets that are activated by at least one ligand combination and gray regions represent subsets that cannot be addressed by any ligand combination. These subsets constitute the “addressing repertoire” of a system. Addressing capability can vary widely. Examples include purely orthogonal activation (top) and all possible subsets (bottom). (B) We optimized parameters to achieve the fully addressable system of (A). Simulating the responses of the three cell types to each ligand word confirms that any of the seven possible subsets can be successfully addressed. (C) We generalized the optimization approach to identify parameters achieving each possible addressing repertoire of three cell types in a mammalian-like model with four type I and three type II receptors. The optimal distinguishability value for each repertoire is plotted. Orange stars indicate addressing repertoires that cannot be achieved in the one-to-one architecture (STAR Methods: Subset addressing in one-to-one model).

• **Distinguishability** (Figures 2, 3, and 4; STAR Methods: Distinguishability of channels) quantifies the specificity of a given addressing scheme and is defined as (lowest on-target activity)/(highest off-target activity). As an example, consider a system where ligand word 1 activates on-target cell type A and off-target cell type B at levels of (0.8, 0.1) units, respectively, while ligand word 2 activates off-target cell type A and on-target cell type B at levels of (0.4, 0.9). The distinguishability for orthogonal addressing of "A" and "B" would then be 0.8/0.4=2. As another example, if cell types A and B are both on-target for ligand word 2, addressing "A" and "AB" would have a distinguishability of 0.4/0.1=4.
• **Addressability** (Figure 6; STAR Methods: Addressability of ligand words) quantifies the diversity of the addressing repertoire for a set of ligand words. We first measure the separation of two ligand words as the largest fold change of their resulting activation levels in any cell type. Addressability is then defined as the separation of the least separable pair of ligand words.

To systematically identify parameters that generate orthogonal channels, we used an optimization approach (Figure 2B). We considered discrete ligand concentrations, allowing each ligand to take on one of three logarithmically spaced concentrations, $10^{0}=1$, $10^{1.5}\approx 32$, and $10^{3}=1,000$ arbitrary units (AU), reflecting the experimentally observed input dynamic range for BMP signaling (Antebi et al., 2017; Bradford et al., 2019; Hatsell et al., 2015). This discretization defines a finite set of $3^{n_{L}}$ possible ligand words. To identify a system with N channels, we chose a subset of N ligand words (Figure 2Bi). Each such choice defines an “addressing scheme.” Achieving an addressing scheme requires identifying N cell types that are each individually activated by one word (Figure 2Bii). We then used least-squares optimization to identify biochemical parameters (affinities, $K_{ijk}$, and activities, $e_{ijk}$) and N receptor expression profiles (one for each cell type) that best implement the target addressing scheme (Figure 2Biii; STAR Methods: Optimization of orthogonal addressing schemes). To obtain a more complete view of the functional behavior, we then computed the responses of each cell type on a higher-resolution (10 × 10) grid of ligand levels (Figure 2Biv).

To quantify the channel structure of the resulting communication system, we computed the crosstalk matrix (Figure 2C), where each row is a ligand word, each column is a cell type, and each value represents the normalized response of that cell type to the corresponding ligand word. Diagonal elements of this matrix represent “on-target” signaling, which ideally approach 1. Off-diagonal elements represent “off-target” signaling, ideally 0.

### Two ligands can orthogonally address five distinct cell types

To test whether the promiscuous architecture can improve on a one-to-one system, we applied our optimization approach to search for two-ligand systems ($n_{L}=2$) that generate three orthogonal channels in a model with two type I and two type II receptor subunits ($n_{A}=2$ and $n_{B}=2$), reflecting the receptor multiplicity seen in *Drosophila*. We enumerated all 31 possible discrete addressing schemes, optimized parameters for each scheme, and analyzed the resulting responses (Figure 2D; STAR Methods: Enumeration of orthogonal addressing schemes). In 28 of the 31 possible schemes, all on-target activity levels (orange shaded regions) exceeded all off-target activity levels (blue shaded regions), giving rise to orthogonal addressing. To quantify the addressing specificity, we computed a distinguishability score, which is defined as the fold difference between the lowest on-target activity and the highest off-target activity (STAR Methods: Distinguishability of channels). The best scheme, based on using each ligand individually as well as a word with both ligands at their maximal level, produced a distinguishability greater than 45 (Figure 2D, scheme 1). (We note that these results represent a lower bound on the potential addressing capacity and specificity, as global optima are not guaranteed.) By contrast, one-to-one systems achieved distinguishability values of only ∼1.4 for three channels (Figure 2D; STAR Methods: Orthogonal addressing in one-to-one model).

Inspection of the addressing schemes showed that they typically used combinations of archetypal response functions previously observed in the BMP signaling pathway (Figure S1) (Antebi et al., 2017). In most schemes, two cell types produced opposite ratiometric responses to the two ligands, with the third cell type exhibiting a variety of other responses (Figure S2). These included a balance detector, in which the combination of the two ligands synergistically activated the pathway more than either ligand alone (Figure S2, e.g., schemes 1 and 2); a nonmonotonic response, in which the pathway was most highly activated at intermediate concentrations of a given ligand (e.g., schemes 3 and 4); a distinct ratiometric response (e.g., schemes 18 and 19); and an additive response to the two ligands (e.g., scheme 17). Many of these response types require ligand-receptor promiscuity. For example, ratiometric responses cannot occur in a one-to-one architecture, as an additional ligand that signals through a different receptor cannot decrease the response to the activating ligand. Thus, the ability of cells to access a variety of multi-ligand response functions with different receptor configurations facilitates addressing.

We extended this analysis to systems with up to eight channels. (The value of eight channels reflects the discretization of ligand concentration space and is not inherent in the system.) With a fly-like model ($n_{L}=2,n_{A}=2,\text{and}\;n_{B}=2$), up to seven orthogonal channels could be addressed with a distinguishability greater than that possible in a corresponding one-to-one model (Figures 3A and 3B; STAR Methods: Orthogonal addressing in one-to-one model). Fewer channels (lower bandwidth) could be achieved with greater distinguishability. For instance, a five-channel scheme exhibited a distinguishability of 3.6 through a combination of ratiometric, balance detection, and nonmonotonic responses (Figure S3A). Taken together, these results demonstrate that two ligands with promiscuous ligand-receptor interactions can address a larger number of cell types, albeit at varying levels of distinguishability.

### Addressing can occur despite gene expression noise

Stochastic fluctuation, or noise, in gene expression presents a challenge for addressing (Elowitz et al., 2002; Raser and O’Shea, 2005). On the one hand, signaling must be sensitive to receptor expression in order for cell types to have different responses to the same ligand words. On the other hand, if sensitivity is too high, receptor expression noise could disrupt addressing. Here, we asked whether addressing could occur despite correlated (extrinsic noise) and uncorrelated (intrinsic noise) fluctuations in receptor expression, each assumed to have a physiologically reasonable coefficient of variation (SD/mean) of 0.5 (Elowitz et al., 2002; Raj et al., 2006; Suter et al., 2011).

To characterize the extent to which each type of noise degrades addressing, we computed receiver operating characteristic (ROC) curves and the corresponding area under the curve (AUC) values (Figure 3C; STAR Methods: Analysis of robustness), which characterize the proportion of on- and off-target cells that are correctly classified (Hanley and McNeil, 1982). (AUC values range from 0.5 for a random system to 1.0 for an ideal system.) The five-channel system showed separation between channels (Figure 3D) but also demonstrated high AUC values of 0.9820 and 0.9400 with extrinsic and intrinsic noise sources, respectively. The more stringent metric of distinguishability, which is sensitive to incorrect activation of even a single cell type, was impacted more by intrinsic than extrinsic noise (Figure S3B, left). These results suggest that minimizing intrinsic noise is important for maximizing addressing capacity.

The addressing capacity of a system depends on the minimum acceptable distinguishability level. Five-channel addressing could be achieved with a distinguishability threshold of 2, which could be physiologically reasonable given that 2-fold changes in signaling pathway activation have been shown to alter cell fate decisions (Dessaud et al., 2007; Falo-Sanjuan et al., 2019; Van de Walle et al., 2013; Zagorski et al., 2017). Greater values of distinguishability, such as 4 or 10, were achieved with four or three channels, respectively (Figure S4). In general, higher distinguishability thresholds translated to reduced noise sensitivity (Figures S4A–S4C). In particular, the three-channel system (distinguishability greater than 10) exhibited AUC values of 0.9992 and 0.9914 for extrinsic and intrinsic noise, respectively. These results show that systems with just two ligands and only two variants of each receptor type can provide multiple channels with reasonable levels of distinguishability.

### The number of addressable channels increases with the number of receptor variants

BMP receptor multiplicity has varied during evolution, leading to different numbers of receptor variants in *Drosophila* (two type I and two type II), humans (four type I and three type II), and other species (Massagué, 1998; Newfeld et al., 1999; O’Connor et al., 2006). What additional addressing capabilities emerge with this increase in receptors? A mammalian-like model with four type I and three type II receptor subunit variants outperformed the fly-like model (Figures 3E and 3F), achieving better specificity at any given number of channels (cf. Figures 3A and 3B). In fact, in this model, two ligands were able to address as many as eight orthogonal channels with a 1.5-fold distinguishability between on- and off-target activity (Figure 3F) and high AUC values (Figure 3G), resulting in a generally diagonal crosstalk matrix (Figure 3H). Six-, five-, and four-channel systems could be achieved with distinguishability values greater than 2, 4, and 10, respectively (Figures S4D–S4F). Five-channel addressing yielded AUCs of 0.9924 and 0.9608 for extrinsic and intrinsic noise, respectively, whereas four-channel addressing gave near-perfect AUCs of 0.9983 and 0.9944 (Figures S4E and S4F).

Eight-channel addressing was more robust to extrinsic than intrinsic noise in receptor expression levels, with AUC values of 0.9794 and 0.8853 for extrinsic and intrinsic noise, respectively (Figure 3G). Distinguishability values remained above 1 for correlated fluctuations, but not for uncorrelated noise, in receptor expression (Figure S3B, right). The overall addressing scheme resulted from diverse single-cell responses, including ratiometric, balance detection, and nonmonotonic behaviors (Figure 3I). Taken together, these results show that a modest increase in the number of receptor variants generates a substantial expansion in addressing capacity, which is achieved through a variety of single-cell responses.

Although eight is the maximum number of channels in the three-level ligand discretization scheme, more channels may be possible with higher-resolution grids. For instance, a four-level ligand discretization scheme allows up to fifteen channels. At this level, it was no longer computationally feasible to systematically test all possible addressing schemes. Instead, we sought to optimize increasing bandwidths, choosing random schemes with a given number of channels until successfully optimized (STAR Methods: Optimization of orthogonal addressing schemes).

We performed this analysis for both the fly-like and mammalian-like models. With a fly-like model, we successfully optimized systems with up to five orthogonal channels (Figures S5A and S5B). This five-channel system exhibited a distinguishability of 2.3 (Figure S5C) and, like the system obtained using a lower-resolution grid, exhibited a combination of ratiometric, balance detection, and nonmonotonic responses (Figure S5D; cf. Figure S3A). In the mammalian-like model, we identified a seven-channel system with a distinguishability of 2.2 as well as a more weakly addressable eight-channel system with a distinguishability of 1.4 (Figures S5E–S5G). The eight-channel system used similar types of responses to those in the three-level discretization scheme (Figure S5H; cf. Figure 3I). Overall, the bandwidths achievable and the responses observed remained qualitatively similar using the higher-resolution ligand grid compared with the three-level discretization. Allowing ligand concentrations to vary continuously or exploring parameter space more comprehensively could reveal a greater addressing capacity.

### Promiscuous architectures enable subset addressing

Combinatorial addressing can extend beyond the addressing of individual cell types, as explored thus far, to generate more complex, multi-cell-type response patterns. In such “subset addressing,” each ligand word activates a specific subset of cell types. In the olfactory system, for example, odorants activate specific subsets of olfactory receptor neurons, giving rise to a combinatorial representation of odors (Hallem and Carlson, 2006; Malnic et al., 1999). Subset addressing systems can be characterized by an “addressing repertoire,” defined as all unique subsets of cell types (channels) that can be addressed across all possible ligand words (Figure 4A). For example, a system with three cell types that can only be orthogonally activated would have three channels (Figure 4A, top). The highest bandwidth of seven addressable subsets occurs when all cell types can be activated in any required combination using some ligand word (Figure 4A, bottom).

We first asked what addressing repertoires are possible in mammalian-like systems with three cell types or seven possible channels. Using the optimization approach, we identified parameters that achieve the fully addressable seven-channel system (Figure 4B; STAR Methods: Optimization of subset addressing repertoires). We then generalized this approach to all 32 possible addressing repertoires (STAR Methods: Enumeration of subset addressing repertoires), successfully identifying a parameter sets that generated every repertoire with a distinguishability greater than 1 and 29 repertoires with a distinguishability greater than 2 (Figure 4C). These results show that two ligand variants can generate any addressing repertoire of three cell types, most with high distinguishability.

Achieving such a broad set of addressing repertoires requires promiscuous ligand-receptor interactions. In a one-to-one model, having high concentrations of all ligands will activate all cell types; therefore, any addressing repertoire in which the three cell types cannot be simultaneously co-activated requires a promiscuous architecture (Figure 4C, orange stars; STAR Methods: Subset addressing in one-to-one model). Taken together, these results demonstrate that the promiscuous ligand-receptor architecture allows diverse addressing repertoires, beyond those achievable in a one-to-one model.

### Cell lines show combinatorial addressing *in vitro*

Having analyzed the theoretical conditions that permit addressing, we next asked whether addressing could occur in living cells. Previous work revealed that individual cell lines exhibit complex responses to ligand combinations that can be altered by perturbing the expression of specific receptors (Antebi et al., 2017). However, it is unclear to what extent the responses generated in this way could allow the differential activation of distinct cell types using different ligand words. Experimentally analyzing the responses of multiple cell lines with differing receptor expression profiles across the same panel of ligand combinations could reveal the potential for addressing.

To this end, we engineered cell lines with different receptor expression profiles and analyzed their responses across two-dimensional titrations of two different ligand pairs—BMP2+BMP9 and BMP9+BMP10—in which combined signaling activity was shown to depend on receptor expression profiles (Klumpe et al., 2022). To read out pathway activity, we used a transcriptional fluorescent reporter for SMAD1/5/8 containing BMP response elements from the Id1 promoter (Korchynskyi and ten Dijke, 2002). We stably integrated the reporter into cell lines with different receptor expression profiles and then analyzed their responses to a range of BMP ligand combinations by flow cytometry 24 h after ligand addition (STAR Methods: Addressing of cell lines). We used ligand concentrations up to 1,000 ng/mL to broadly survey physiologically relevant levels. Although measured serum levels are around 0.1–10 ng/mL (Albilia et al., 2013; David et al., 2008; Herrera and Inman, 2009; Penn et al., 2017), effective levels at the cell surface are likely to be higher due to local production, consistent with the higher concentrations of 10–500 ng/mL used for *in vitro* studies of various BMP-dependent processes (Blackwell et al., 2009; Grassinger et al., 2007; Kim et al., 2013; LaVaute et al., 2009; Valera et al., 2010; Zhao et al., 2003; Zhu et al., 2017). Finally, BMP ligands are used clinically in concentrations on the order of 1,000 ng/mL (Gupta and Khan, 2005; Kim et al., 2015). Thus, our chosen titration range effectively represents these varied conditions.

**Figure 5:**
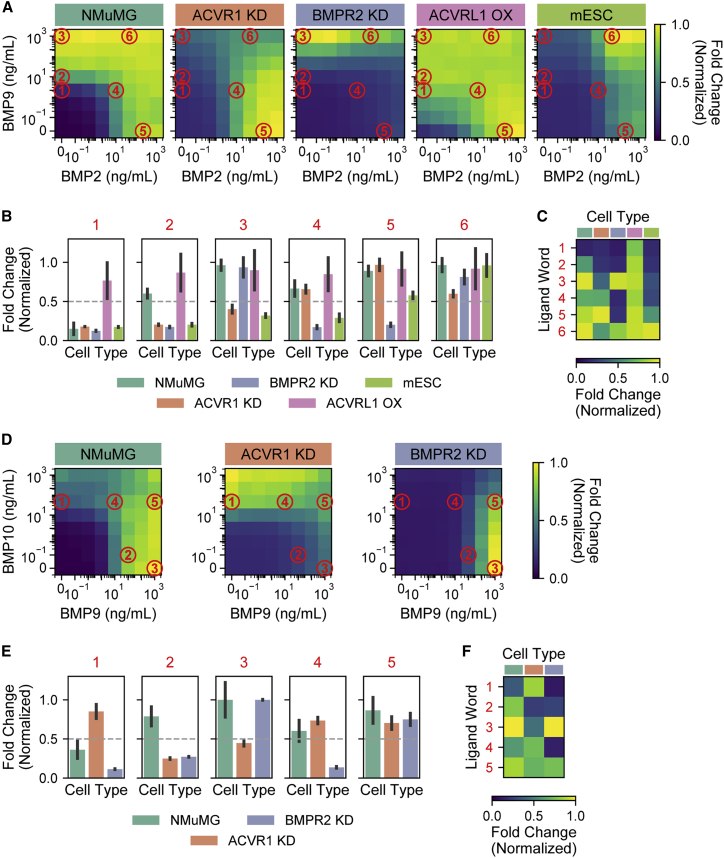
Cell lines preferentially respond to different ligand combinations (A) Responses were measured for, from left to right, NMuMG cells, NMuMG cells with ACVR1 knockdown (KD), NMuMG cells with BMPR2 KD, NMuMG cells with ACVRL1 overexpression (OX), and mESCs, using flow cytometry of an integrated fluorescent protein reporter (STAR Methods: Addressing of cell lines). Each cell line was exposed to a double titration of BMP2 and BMP9, and responses were quantified by taking the mean of at least 3 replicates. For each cell line, fold change is calculated relative to the baseline fluorescence with no added ligand and then normalized by the maximum value. Responses at select ligand words (red circles) are analyzed further in (B). (B) For select ligand words from (A), the responses of each cell line are shown. Error bars indicate SD of at least 3 repeats. Ligand words were chosen by fixing a threshold of 0.5 (gray dashed line) and identifying those ligand combinations yielding unique on- and off-target activation patterns. (C) Data from (B) are summarized by showing the response of each cell type (columns) to each ligand word (rows), illustrating that distinct ligand words can activate different subsets of cell types. (D) Responses of NMuMG, NMuMG with ACVR1 KD, and NMuMG with BMPR2 KD to BMP9 and BMP10 are shown, as in (A). (E) As in (B), the responses of each cell type at selected ligand words are shown. (F) As in (C), the responses of each cell type (columns) to each ligand word (rows) confirm that distinct ligand words preferentially activate distinct groups of cell types. See also Table S1.

We started with a previously characterized epithelial cell line, NAMRU mouse mammary gland (NMuMG) cells, that robustly responds to a variety of BMP ligands (Antebi et al., 2017). NMuMG cells responded additively to BMP2 and BMP9 (Figure 5A, first), consistent with previous results (Antebi et al., 2017). Since opposing ratiometric responses are a common “motif” in the addressing schemes identified above (Figures 2 and 3), we sought to generate additional cell lines that would exhibit such responses by knocking down receptors with known preferences for each specific ligand. In the NMuMG background, the knockdown of ACVR1, which directly interacts with BMP9 (Luo et al., 2010), resulted in a minimal response to BMP9 but a strong response to BMP2, thereby generating a ratiometric response profile (Figure 5A, second). By contrast, the knockdown of BMPR2, the major BMP2 receptor (Xia et al., 2007), gave rise to a reduced responsiveness to BMP2 with a strong BMP9 response, producing a complementary ratiometric response (Figure 5A, third). In contrast to receptor knockdown, which increases competition for a limited receptor pool and, therefore, could increase the complexity of multi-ligand responses, we anticipated that ectopic receptor expression might relieve receptor competition and thereby generate more additive responses (Klumpe et al., 2022). Indeed, ectopic ACVRL1 expression increased the sensitivity to ligands without qualitatively altering the combinatorial response (Figure 5A, fourth). Finally, for comparison with a more distantly related cell type, we also analyzed E14 mouse embryonic stem cells (mESCs), which differ in receptor expression profile from NMuMG cells in at least three receptors (Antebi et al., 2017). mESCs responded maximally to combinations of BMP2 and BMP9 together (Figure 5A, fifth).

Comparing the responses of these cell lines across double titrations of BMP2 and BMP9 showed that the two ligands could be used at different concentrations to preferentially activate certain cell types individually or in groups (Figure 5B). For example, moderate levels of BMP9 alone predominantly activated NMuMG with ectopic ACVRL1 (Figure 5B, word 1), whereas higher levels additionally activated first NMuMG (word 2) and then NMuMG with BMPR2 knockdown (word 3). Intermediate levels of both ligands abolished the activation of NMuMG with BMPR2 knockdown and allowed the activation of NMuMG with ACVR1 knockdown (word 4). High levels of BMP2 activated all cell types except NMuMG with BMPR2 knockdown (word 5), and high levels of both ligands activated all cell types (word 6). In this way, distinct combinations of BMP2 and BMP9 enabled preferential activation of six distinct combinations of five cell types (Figure 5C), establishing that the BMP pathway has combinatorial addressing capability.

To extend this analysis to another ligand pair, we evaluated the responses of three NMuMG-derived lines to BMP9 and BMP10 (Figure 5D). In this pairwise titration, NMuMG and NMuMG with BMPR2 knockdown responded more strongly to BMP9 than to BMP10 alone, with BMP10 reducing the activation by BMP9 when present in combination. BMP10 inhibition of BMP9 signaling was stronger when BMPR2 was knocked down. By contrast, ACVR1 knockdown cells exhibited the opposite response, responding more strongly to BMP10 than to BMP9. We were able to identify five ligand words that activated distinct combinations of these three cell types (Figures 5E and 5F). BMP10 alone activated only ACVR1 knockdown cells (Figure 5E, word 1). Intermediate levels of BMP9 activated the wild-type cells (word 2), whereas higher levels additionally activated BMPR2 knockdown cells (word 3). NMuMG and ACVR1 knockdown cells could be simultaneously activated with intermediate levels of both ligands (word 4), whereas additional BMP9 enabled the activation of all three cell types simultaneously (word 5). These results provide additional evidence that the BMP pathway could potentially support addressing.

The ability to achieve addressing *in vitro* does not demonstrate that addressing occurs in physiological contexts. However, single-cell gene expression atlases reveal that the receptor profiles of NMuMG cells and their perturbed derivatives resemble those in some natural cell types (Table S1) (Tabula Muris Consortium, 2020; Tabula Muris Consortium et al., 2018). It will be interesting to determine whether the profiles analyzed here play natural addressing roles *in vivo*.

### Response function diversity increases addressability

The values of key biochemical parameters—affinities and activities—ultimately determine the addressing bandwidth of a promiscuous ligand-receptor system. What is the distribution of addressing bandwidth across different parameter sets? Are there design rules that allow the tuning of those values, in absolute or relative terms, to optimize addressing? Information theory provides a natural framework to answer these questions (Huntley et al., 2016; Itzkovitz et al., 2006). More specifically, the concept of mutual information can be used to quantify the addressing power of a promiscuous ligand-receptor system without assuming any particular choice of ligand words or cell types or any particular mapping between them (STAR Methods: Computation of mutual information).

**Figure 6:**
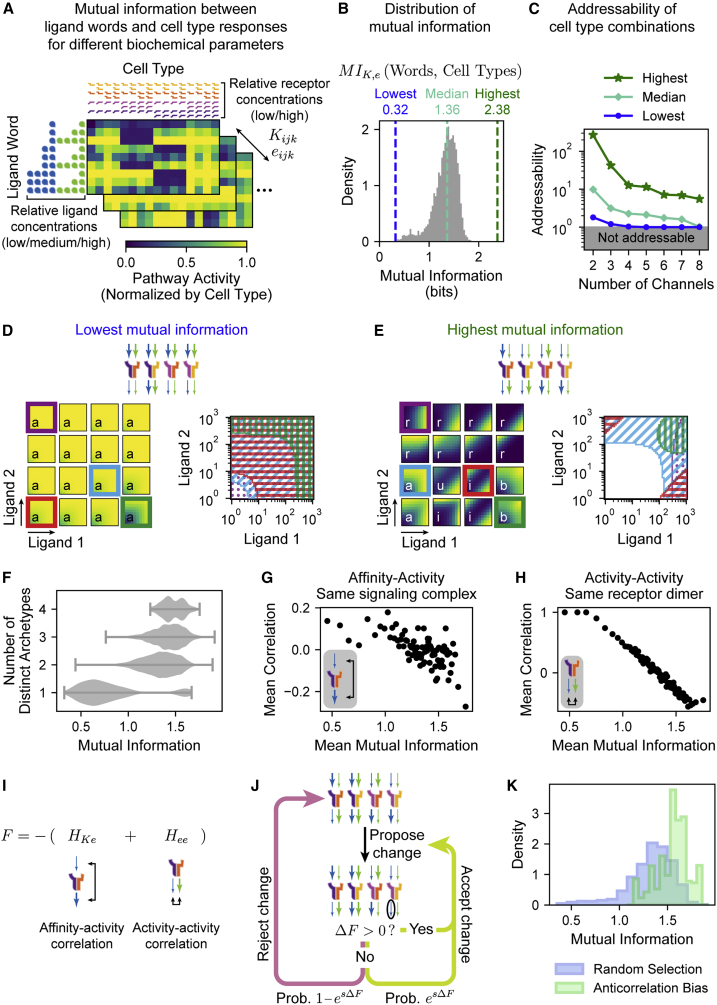
Information theoretic analysis reveals design principles for combinatorial addressing (A) Mutual information between a comprehensive library of ligand words (rows) and the corresponding activation patterns across a library of cell types (columns) can be computed across a systematic grid-based sampling of the biochemical parameters K and e (matrices). For each row, one, two, and four ligand symbols indicate low (10^0^), medium (10^1.5^), or high (10^3^) concentrations of the indicated ligand. Similarly, one or two receptor symbols indicate low (1) or high (100) levels of the indicated receptor for each column. (B) The distribution of mutual information across biochemical parameters is shown. Dashed lines indicate the lowest (blue), median (cyan), and highest (green) values. High mutual information indicates that many distinct cell-type combinations can be specifically activated by distinct ligand words. (C) The addressability values of the activated subsets are shown for different numbers of channels. The addressability reflects the minimal fold difference in the response of at least one cell type when exposed to any two distinct ligand words (STAR Methods: Addressability of ligand words). Results are shown for three sets of biochemical parameters generating the lowest, median, and highest mutual information values. (D) The parameter set with the lowest mutual information is represented schematically (top), as in Figure 2Biii. For these parameters, the responses for the library of 16 cell types are shown as a 4 × 4 grid (bottom left). In each response, the x and y axes represent logarithmic titrations of ligands 1 and 2, respectively. All show the same qualitative response of additive (“a”) behavior, differing only in their quantitative sensitivity. Schematically, overlaying four differing responses (highlighted in purple, cyan, red, and green) reveals that different ligand words largely address similar combinations of cell types (bottom right), with relatively few distinct subsets represented. (E) For the parameter set with the highest mutual information (top), the cell types in the library show a variety of response patterns (bottom left): ratiometric (“r”), additive (“a”), imbalance (“i”), and balance (“b”), matching the response archetypes (Figure S1) previously observed experimentally (Antebi et al., 2017). One response not fully matching any archetype is unclassified (“u”). Schematically, overlaying four differing responses (purple, cyan, red, and green) reveals that different ligand words can address many distinct subsets of cell types (bottom right). Note that complexes tend to have opposite values of affinity and activity parameters as well as other parameter anticorrelations, as analyzed in (G) and (H). (F) Violin plots indicate the distribution of mutual information values for systems with different numbers of distinct archetypes represented among individual cell response functions. Note that greater archetype diversity enriches for high mutual information. (G) Anticorrelation of affinity and activity parameters for the same complex is associated with higher mutual information. We analyzed average properties across bins of 800 parameter sets. To measure the correlation between affinity and activity of complexes, we represented low and high values as -1 and 1 and computed the dot product between K and e vectors. The average correlation and mutual information across bins are plotted. (H) Parameter sets with high mutual information show anticorrelation in the activities of complexes with the same receptor but different ligands. Analysis was done analogous to (G). (I) We defined a fitness function F that rewards parameter sets exhibiting the anticorrelations observed in (G) and (H). (J) An evolutionary algorithm identifies parameter sets that maximize F. At each iteration, a random parameter value is flipped from low to high or vice versa. Changes that increase F are accepted. Changes that decrease F are accepted with indicated probability (bottom), which depends on a selection pressure parameter s. This process is repeated iteratively (STAR Methods: Evolutionary algorithm as a generative model). (K) An evolutionary algorithm enriches for high mutual information. We ran the algorithm with s>0 to favor anticorrelations or with s=0 to randomly sample parameters. For each case, we randomly initialized 2,000 parameter sets and performed 200 iterations. We then evaluated the mutual information for the final value of the parameter set and visualized the resulting distributions. Random selection (s=0, blue) led to a similar distribution of values as the systematically sampled parameter sets (cf. Figure 6B), whereas favoring anticorrelations (s>0, green) resulted in an overall increase in mutual information. See also Figure S6.

To identify parameter sets that maximize mutual information, we systematically analyzed the diversity of responses across a set of cell types to a set of ligand words for different biochemical parameter sets (Figure 6A). Mutual information measures information communicated by the optimal subset of ligand words to the optimal subset of cell types, allowing the use of comprehensive libraries. In a fly-like model, we constructed a discrete ligand word library in which each of two ligands takes on one of three concentration values ($3^{n_{L}}$ ligand words, or 9): a cell type library in which each of the two type I and two type II receptors is expressed at one of two values ($2^{n_{A}+n_{B}}$ cell types, or 16) and a biochemical parameter library in which each $K_{ijk}$ and $e_{ijk}$ takes on one of two values ($2^{2n_{L}n_{A}n_{B}}$ parameter sets, or 65,536). We then simulated the response of each cell type to each ligand word for each biochemical parameter set and computed the mutual information between the sets of ligand words and pathway activities across the library of cell types (Figures 6A and 6B; STAR Methods: Analysis of mutual information). Random, rather than grid-based, sampling of $K_{ijk}$ and $e_{ijk}$ produced similar results (Figure S6A). Mutual information values varied broadly across parameter sets, from 0.32 to 1.91 bits, with a median value of 1.36 bits (Figure 6B). By refining our search over biochemical parameters, we were able to identify parameters with values as high as 2.38 bits (STAR Methods: Optimization of mutual information).

To assess whether mutual information correlates with addressing, we defined an addressability metric, which quantifies how strongly activation patterns differ for different ligand words without requiring specific targeted profiles (STAR Methods: Addressability of ligand words). For every pair of ligand words, we identified the largest fold difference of activation levels across all cell types. This value is high when two ligand words induce distinct responses in at least one cell type. We defined the addressability metric as the lowest such value across all ligand word pairs and calculated this value for a given number of channels N by taking the best choice of all possible subsets of N ligand words. Using this metric, we analyzed addressability for systems with low, intermediate, and high mutual information (Figure 6C). For the parameter set with highest mutual information (2.38 bits), each of the eight ligand words activated a distinct cell-type combination with over 5.5-fold addressability. The median parameter set (1.36 bits) addressed up to seven distinct cell-type combinations at an addressability of 1.6, whereas the parameter set with lowest mutual information (0.32 bits) addressed only three distinct cell-type combinations with addressability of 1.2. Overall, a 1-bit difference in mutual information can increase addressing specificity as well as bandwidth, enabling diverse responses to different ligand words.

We next sought to understand how high addressing bandwidth arises from the individual response functions of each cell type by comparing the parameter sets with the lowest and highest mutual information. The parameter set with the lowest mutual information generated a homogeneous spectrum of responses across all cell types (Figure 6D; STAR Methods: Analysis of archetypal responses). These responses predominantly varied quantitatively in their sensitivity to ligand. By failing to fully exploit the two-dimensional nature of ligand concentration space, this parameter set exhibited limited addressing potential. By contrast, the parameter set with the highest mutual information generated a broad diversity of ligand response functions across the cell types, reproducing the experimentally observed ratiometric, additive, imbalance detection, and balance detection “archetypal” functions (Figure 6E; STAR Methods: Analysis of archetypal responses) (Antebi et al., 2017). By generating diverse two-dimensional response functions, this parameter set allowed each ligand word to activate a distinctive combination of cell types. In fact, such a correlation between the diversity of response functions and mutual information is seen across the full library of parameter sets (Figure 6F).

### Affinity-activity relationships control addressing bandwidth

How do parameter sets with high mutual information generate the varied response functions associated with addressing? Inspection of the parameter set with the highest mutual information revealed two notable relationships between binding affinities $K_{ijk}$ and signaling efficiencies $e_{ijk}$ (Figure 6E). First, complexes that formed with strong affinity (large $K_{ijk}$) often had low signaling efficiency (small $e_{ijk}$). Second, the activity of a given receptor pair strongly depended on the identity of the bound ligand, producing opposite values for $e_{1jk}$ or $e_{2jk}$. Systematic analysis of these relationships revealed their dependence on mutual information (STAR Methods: Analysis of parameter correlations). In particular, anticorrelations between the affinity and activity ($K_{ijk}$ and $e_{ijk}$; Figure 6G) and between the activities of complexes with distinct ligands ($e_{1jk}$ and $e_{2jk}$; Figure 6H) were associated with higher mutual information. These results suggest that such anticorrelations could predict high addressing capacity.

To test whether the anticorrelated structure of the parameters is sufficient to produce high mutual information, we developed an evolutionary algorithm that evolves the biochemical parameters to maximize the above anticorrelations (Figures 6I–6K). The algorithm starts with an initial parameter set, proposes a random change to one $K_{ijk}$ or $e_{ijk}$ value, and accepts that change with probability 1 if the change increases the fitness function F and with probability $e^{s\Delta F}$ if it does not, where s is a parameter that controls the strength of the selection (STAR Methods: Evolutionary algorithm as a generative model). Iteration of this procedure increased mutual information between ligand words and cell-type responses to values comparable with the strongest ones identified in the systematic screen (Figure 6K; cf. Figure 6B).

These results indicate that strong addressing is not rare. It is realized to varying degrees across all of parameter space and enhanced by specific parameter anticorrelations. Further, experimental analysis of ligand-receptor interactions suggests that the natural BMP system may exhibit such anticorrelations (Klumpe et al., 2022). When systematic measurements of responses to pairwise ligand combinations were fit to the same model used here, four of the five ligands analyzed (BMP4, BMP7, BMP9, and BMP10) exhibited properties consistent with the formation of strong-affinity, low-activity complexes (e.g., with the BMPR1A/ACVR2B receptors) and weak-affinity, high-activity complexes (e.g., with the ACVR1/ACVR2A receptors). Overall, the parameter fits from this study are consistent with a broad range of addressing capabilities (Figure S6B).

## Discussion

Communication systems such as email enable one to address messages to specific recipients and groups of recipients. Similarly, in multicellular organisms, it is crucial to activate the right cells at the right time and place. A fundamental mystery in cell-cell communication is how freely diffusing ligands can precisely target, or address, specific cell types. The promiscuity of ligand-receptor interactions in BMP and other communication pathways makes this question especially perplexing, since it appears to reduce rather than enhance communication specificity. However, promiscuous architectures are employed for specificity in other biological contexts. For example, promiscuous ligand-receptor interactions in the olfactory system enable a limited number of receptors to sense a great diversity of odorants through a combinatorial population code (Duchamp-Viret et al., 1999; Goldman et al., 2005; Hallem and Carlson, 2006; Malnic et al., 1999). Such architectures also appear analogous to simple neural networks, which can compute complex functions of multi-dimensional inputs (Bray, 1995). This computational ability could allow different cell types to respond to different ligand combinations, as observed experimentally (Figure 5) (Antebi et al., 2017; Klumpe et al., 2022).

**Figure 7:**
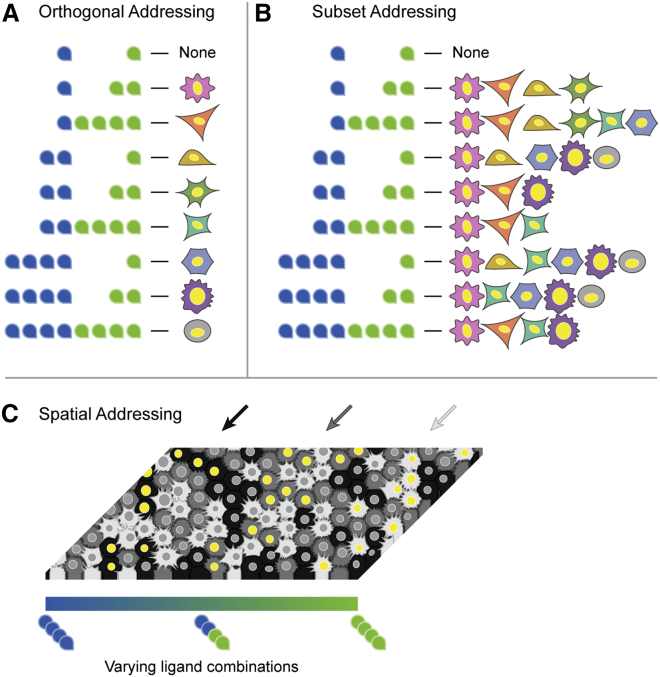
Promiscuous ligand-receptor interactions allow flexible, high-bandwidth addressing (A) Promiscuous ligand-receptor interactions enable orthogonal addressing in which individual cell types can be specifically activated using combinations of only two different ligand variants (cf. Figures 3E–3I). (B) Promiscuous ligand-receptor interactions enable subset addressing in which different ligand words address diverse cell-type combinations (cf. Figure 6E). (C) This notional schematic shows how two antiparallel morphogen gradients could address different cell types (black, dark gray, and light gray) in specific spatial regions. Yellow nuclei indicate activation. In this example, high levels of blue ligand activate the black cell type (left), the combination of both ligands (blue and green) activates the dark gray cell type (center), and high levels of green ligand activate the light gray cell type (right).

Our results show that promiscuity could potentially allow ligand combinations to address different cell types or groups of cell types with remarkable specificity (Figures 7A and 7B). Compared with one-to-one architectures that achieve perfect specificity for a limited number of channels, promiscuous signaling pathways can target a large number of cell types at higher specificity (Figure 3) as well as enable greater flexibility in addressing arbitrary subsets of cell types (Figure 4). High addressing capacity can be a robust feature of promiscuous ligand-receptor systems, withstanding correlated noise in receptor expression levels (Figures 3C and 3G) and emerging across a broad range of biochemical parameter values. A more general mutual information framework identified design principles that maximize addressing capacity (Figure 6). In particular, these include anticorrelation between the affinity and activity of a given ligand-receptor complex and anticorrelation between the activities of two ligands interacting with the same receptor dimer. Together, these results show how addressing specificity can emerge from molecular promiscuity in a canonical cell-cell communication system.

Are the biochemical parameters of the natural BMP pathway compatible with addressing? Quantification of BMP ligand-receptor interaction parameters has been done for select components, although direct measurements of the activity of specific signaling complexes have not yet been achieved (Karim et al., 2021). Systematic analysis of pairwise ligand combinations showed complex responses to ligand combinations and revealed their dependence on specific receptors (Klumpe et al., 2022). When fit to the same model used here (Figure 2A), these data provide estimated values for $K_{ijk}$ and $e_{ijk}$. These values exhibit the types of anticorrelations that favor addressing, suggesting that the BMP pathway may have evolved to facilitate high-capacity addressing. However, experimental studies of specific developmental systems will be necessary to establish to what extent combinatorial addressing functions in natural contexts.

The addressing principles described here do not reflect the full complexity of cellular signaling systems. We have focused on computations that can arise through promiscuous ligand-receptor binding and activation of intracellular second messengers. However, our model omits myriad additional processes that can modulate and regulate cell signaling. Examples include nonhomogeneous spatial distribution of ligands and receptors, such as in polarized cells (DeWitt et al., 2002; Kuwada et al., 1998); trafficking of receptors (Burke et al., 2001; Resat et al., 2003; Shankaran et al., 2012) and second messengers (Schmierer et al., 2008); and signaling-induced feedback loops (Shankaran et al., 2007). These processes can enrich the complexity of computations beyond those analyzed here and thereby potentially further enhance the number and distinguishability of addressing channels. Although smaller, more focused models have been essential for developing our current understanding of signaling, fully describing biological systems will require building integrated models that span multiple scales and processes (Wells and Wiley, 2018; Wiley et al., 2003).

BMPs function as morphogens, provoking the question of whether and how addressing plays out in a dynamic, spatially extended “heterocellular” tissue context (Wells and Wiley, 2018). BMP-dependent developmental patterning processes typically use multiple BMP ligands in spatially and temporally overlapping gradients that can be further shaped by shuttling and other extracellular processes. For example, during early *Xenopus* embryo development, an antiparallel gradient of BMP ligands is formed between ventral and dorsal centers (Ben-Zvi et al., 2008; Reversade and De Robertis, 2005). Similarly, overlapping expression patterns of GDF5 and multiple BMP ligands, together with distinct receptor expression patterns, play a key role in the activation (and suppression) of BMP signaling in specific cell populations during joint formation (Lyons and Rosen, 2019; Salazar et al., 2016). In such overlapping gradients, addressing could allow different cell types, all with functional BMP pathways, to each selectively respond in distinct regions based on the concentrations of multiple ligands (Figure 7C).

Additionally, temporal changes in receptor expression are common during development (Danesh et al., 2009; Dewulf et al., 1995; Erickson and Shimasaki, 2003; Sanyal et al., 2002). For instance, in neural precursors, *Bmpr1a* is expressed early and ubiquitously; subsequent treatment with BMP2 induces activation of BMPR1A and expression of *Bmpr1b* (Panchision et al., 2001). These different receptor expression states could preferentially respond to different ligand combinations and, therefore, be addressable. Spatiotemporal addressing could be tested experimentally by genetically modifying the expression of BMP variants in developmental contexts and analyzing the effects on different cell types. *In vitro* reconstitution of multi-ligand gradients could allow a complementary, systematic analysis of spatial addressing (Li et al., 2018).

An increasing amount of receptor expression data is available from cell atlas projects. Together with quantitative measurements of effective biochemical parameters, these data could potentially be used to design ligand combinations that selectively address target cell populations. The ability to design selective targeting would be useful in biomedical applications such as directed differentiation and targeted therapy. For example, recombinant BMP2 has been tested in a variety of therapeutic applications, which are largely related to promoting bone healing and regrowth. However, there are substantial risks, such as ectopic bone formation, respiratory failure, tissue inflammation, and others (Epstein, 2011; Poon et al., 2016). If these complications result from the undesired activation of off-target cell types, using a combination of ligands could potentially provide more specific addressing of the appropriate cell type(s). Other potential therapeutic applications for modulators of BMP signaling include cardiac fibrosis, where both BMP2 and BMP7 have shown promise in animal models (Flevaris et al., 2017; Wang et al., 2012); Parkinson disease, where both BMP2 and GDF5 appear to promote the survival of dopaminergic neurons (Hegarty et al., 2014; O’Keeffe et al., 2017; O’Sullivan et al., 2010); and cancer, where the inhibition of BMP signaling reduces tumor formation in mice (Yokoyama et al., 2017). As the range of clinical applications targeting BMP signaling continues to grow, it will be essential to determine whether combinations of ligands could provide greater specificity than individual ligands.

The principles elucidated here in the context of BMP signaling could apply to other pathways that exhibit promiscuous ligand-receptor interactions, including the broader TGF-β pathway as well as the Wnt, Eph-Ephrin, FGF, and JAK-STAT pathways. The principle of addressing suggests that beyond sensing the concentration of a given set of ligands, these pathways may serve more broadly as computational devices that exploit promiscuous interactions, enabling cells to tune in to specific ligand words and thereby receive information specifically addressed to them.

## STAR★Methods

### Key resources table

**Table:** 

REAGENT or RESOURCE	SOURCE	IDENTIFIER
**Chemicals, Peptides, and Recombinant Proteins**		

BMP2	R&D Systems	Cat# 355-BM
BMP9	R&D Systems	Cat# 5566-BP
BMP10	R&D Systems	Cat# 6038-BP
Fetal Bovine Serum	Clontech	Cat# 631367
Fetal Bovine Serum, ES Cell Qualified	Gibco	Cat# 16141
Leukemia Inhibitory Factor	MilliporeSigma	Cat# ESG1107
Trypsin	Gibco	Cat# 25200
Accutase	Gibco	Cat# A11105

**Deposited Data**		

Optimization and simulation results	This paper	https://doi.org/10.22002/D1.1692
Experimental measurements of BMP responses in multiple cell lines	This paper	https://doi.org/10.22002/D1.1692

**Experimental Models: Cell Lines**		

NMuMG sensor line	Klumpe et al., 2022	N/A
NMuMG sensor line with ACVR1 knockdown	Klumpe et al., 2022	N/A
NMuMG sensor line with BMPR2 knockdown	Klumpe et al., 2022	N/A
NMuMG sensor line with ACVRL1 overexpression	Klumpe et al., 2022	N/A
mESC sensor line	Antebi et al., 2017	N/A

**Software and Algorithms**		

MATLAB	MathWorks	N/A
Python	Python Software Foundation	N/A
Equilibrium Toolkit (EQTK) (Python)	Bois, 2020	https://github.com/justinbois/eqtk and https://doi.org/10.22002/D1.1430
PromiSys (Python)	This paper	https://github.com/christinasu/PromiSys and https://doi.org/10.22002/D1.20047
Simulation and analysis code (Python)	This paper	https://github.com/christinasu/PromiSys and https://doi.org/10.22002/D1.20047
EasyFlow (MATLAB)	Antebi et al., 2017	https://github.com/AntebiLab/easyflow

### Resource availability

#### Lead contact

Further information and requests for resources and reagents should be directed to and will be fulfilled by the lead contact, Michael B. Elowitz (melowitz@caltech.edu).

#### Materials availability

This study did not generate new unique reagents.

### Experimental model and subject details

#### Cell lines

NAMRU mouse mammary gland (NMuMG) cells (female) were acquired from ATCC (CRL-1636). Mouse embryonic stem cells (mESCs; E14Tg2a.4, male) were obtained from the laboratory of Bill Skarnes and Peri Tate. Reporter cell lines were generated as in (Klumpe et al., 2022) and cultured as in (Antebi et al., 2017). Briefly, cells were cultured in a humidity-controlled chamber at 37°C with 5% CO_2_. NMuMG cells were cultured in DMEM supplemented with 10% FBS (Clontech #631367), 1 mM sodium pyruvate, 1 unit/mL penicillin, 1 μg/mL streptomycin, 2 mM L-glutamine, and 1× MEM nonessential amino acids. mESCs were plated on tissue culture plates pre-coated with 0.1% gelatin and cultured using DMEM supplemented with 15% FBS (Gibco #16141), 1 mM sodium pyruvate, 1 unit/mL penicillin, 1 μg/mL streptomycin, 2 mM L-glutamine, 1× MEM nonessential amino acids, 55 mM β-mercaptoethanol, and 1000 units/mL leukemia inhibitory factor (LIF).

### Method details

#### One-step model for promiscuous interactions

##### Ligand-receptor interactions

Many signaling pathways demonstrate promiscuous interactions between multiple ligand and receptor variants, which can bind with varying affinities to form many distinct signaling complexes. The BMP pathway represents a canonical example of such an architecture. Previously, we have described a mathematical model that captures key features of this pathway and recapitulates experimentally observed responses (Antebi et al., 2017). Here, we develop a simplified version of the model that captures equivalent behaviors at steady state while reducing the number of parameters to be considered.

In the model, we describe binding of a ligand to a heterodimer of type I and type II receptors. Specifically, we consider $n_{L}$ ligand variants, $n_{A}$ type I or A receptor variants, and $n_{B}$ type II or B receptor variants, where ligand $L_{i}$ can interact with A receptor $A_{j}$ and B receptor $B_{k}$ to form the heterotrimeric signaling complex $T_{ijk}$. We assume that this process occurs as a one-step reaction with an effective three-way interaction, with forward rate $k_{f_{ijk}}$ and reverse rate $k_{r_{ijk}}$. This reaction can be summarized as

(Equation 1) $$L_{i}+A_{j}+B_{k}\begin{array}{c} k_{f_{ijk}}\\ ⇌\\ k_{r_{ijk}} \end{array}T_{ijk}$$

##### Differential equations and constraints

Letting $L_{i}$ denote the concentration of ligand in a volume V and letting $A_{j}$, $B_{k}$, and $T_{ijk}$ denote the absolute numbers of receptors and complexes on the cell surface, we can then write the differential equations that describe the dynamics of these reactions:

(Equation 2) $$\frac{dL_{i}}{dt}=\frac{1}{V}\sum_{j=1}^{n_{A}}\sum_{k=1}^{n_{B}}\left(-k_{f_{ijk}}L_{i}A_{j}B_{k}+k_{r_{ijk}}T_{ijk}\right)$$

(Equation 3) $$\frac{dA_{j}}{dt}=\sum_{i=1}^{n_{L}}\sum_{k=1}^{n_{B}}\left(-k_{f_{ijk}}L_{i}A_{j}B_{k}+k_{r_{ijk}}T_{ijk}\right)$$

(Equation 4) $$\frac{dB_{k}}{dt}=\overset{n_{L}}{\underset{i=1}{\sum }}\overset{n_{A}}{\underset{j=1}{\sum }}\left(-k_{f_{ijk}}L_{i}A_{j}B_{k}+k_{r_{ijk}}T_{ijk}\right)$$

(Equation 5) $$\frac{dT_{ijk}}{dt}=k_{f_{ijk}}L_{i}A_{j}B_{k}-k_{r_{ijk}}T_{ijk}$$

Each complex $T_{ijk}$ phosphorylates the second messenger at some rate $\varepsilon_{ijk}$ to generate intracellular signal S, which degrades at rate γ. The rate of change of the total signal is given by the following differential equation:

(Equation 6) $$\frac{dS}{dt}=\sum_{i=1}^{n_{L}}\sum_{j=1}^{n_{A}}\sum_{k=1}^{n_{B}}\varepsilon_{ijk}T_{ijk}-\gamma S$$

We assume that the volume for the ligands is large, or V→∞. In this regime, there are significantly more ligand molecules than receptors, as is the case for experimental conditions in which ligands are dissolved in an excess of media. Under this assumption, ligand concentrations remain constant. We further assume that production and consumption of the various molecular species are in steady state. By conservation of mass, the total number of each type of molecule, alone or in complex with other species, must remain constant. Letting $L_{i}^{0}$, $A_{j}^{0}$, and $B_{k}^{0}$ denote the initial values of the respective species, we obtain the following constraints:

(Equation 7) $$L_{i}^{0}=L_{i}$$

(Equation 8) $$A_{j}^{0}=A_{j}+\sum_{i=1}^{n_{L}}\sum_{k=1}^{n_{B}}T_{ijk}$$

(Equation 9) $$B_{k}^{0}\;=B_{k}+\sum_{i=1}^{n_{L}}\sum_{j=1}^{n_{A}}T_{ijk}$$

##### Steady-state equations

Since binding and unbinding of ligands and receptors occur on fast time scales relative to the time scales of reporter detection, we focus on characterizing the behavior of this system at steady state. Here, all time derivatives in Equations 2, 3, 4, 5, and 6 vanish. Defining affinities $K_{ijk}\equiv k_{f_{ijk}}/k_{r_{ijk}}$and activities $e_{ijk}\equiv \varepsilon_{ijk}/\gamma$, Equations 5 and 6 can be solved as follows:

(Equation 10) $$T_{ijk}=K_{ijk}L_{i}A_{j}B_{k}$$

(Equation 11) $$S=\sum_{i=1}^{n_{L}}\sum_{j=1}^{n_{A}}\sum_{k=1}^{n_{B}}e_{ijk}T_{ijk}$$

Together, Equations 7, 8, 9, 10, and 11 describe the behavior of the model at steady state.

$$L_{i}^{0}=L_{i}$$

$$A_{j}^{0}=A_{j}+\sum_{i=1}^{n_{L}}\sum_{k=1}^{n_{B}}T_{ijk}$$

$$B_{k}^{0}\;=B_{k}+\sum_{i=1}^{n_{L}}\sum_{j=1}^{n_{A}}T_{ijk}$$

$$T_{ijk}=K_{ijk}L_{i}A_{j}B_{k}$$

$$S=\sum_{i=1}^{n_{L}}\sum_{j=1}^{n_{A}}\sum_{k=1}^{n_{B}}e_{ijk}T_{ijk}$$

We can solve this system of equations to find the values of $T_{ijk}$ at steady state, which we can then use to compute the total signal S. From Equations 8 and 9, the steady-state values of the receptors are as follows:

(Equation 12) $$A_{j}=A_{j}^{0}-\sum_{i=1}^{n_{L}}\sum_{k=1}^{n_{B}}T_{ijk}$$

(Equation 13) $$B_{k}=B_{k}^{0}-\sum_{i=1}^{n_{L}}\sum_{j=1}^{n_{A}}T_{ijk}$$

Substituting into Equation 10, we have a system of $n_{T}=n_{L}n_{A}n_{B}$ quadratic equations for $T_{ijk}$:

(Equation 14) $$T_{ijk}=K_{ijk}L_{i}\left(A_{j}^{0}-\sum_{i^{\prime }=1}^{n_{L}}\sum_{k^{\prime }=1}^{n_{B}}T_{i^{\prime }jk^{\prime }}\right)\left(B_{k}^{0}-\sum_{i^{\prime }=1}^{n_{L}}\sum_{j^{\prime }=1}^{n_{A}}T_{i^{\prime }j^{\prime }k}\right)$$

The solutions for $T_{ijk}$ from this system of equations can then be substituted into Equation 11 to compute the total signal S.

To solve the model efficiently, we used Equilibrium Toolkit (EQTK), an optimized Python-based numerical solver for biochemical reaction systems (Bois, 2020). EQTK casts the coupled equilibrium problem as an unconstrained convex dual optimization problem and employs a globally convergent trust region algorithm to solve it (Bois, 2020; Dirks et al., 2007). This method accelerated computation by approximately 600-fold compared to standard nonlinear least-squares optimization used previously (Antebi et al., 2017).

#### Comparison with alternative models

##### Promiscuous vs. one-to-one model

We compare a promiscuous signaling architecture to a simple model for signaling with one-to-one ligand-receptor interactions, where each ligand variant $L_{i}$ binds with a single cognate receptor $R_{i}$ to form an active dimer $D_{i}$. We can derive the equations describing this system analogous to the analysis done above for the promiscuous architecture. Assuming that this binding has forward rate $k_{f_{i}}$ and reverse rate $k_{r_{i}}$, the chemical reactions can be expressed as follows:

(Equation 15) $$L_{i}+R_{i}\begin{array}{c} k_{f_{i}}\\ ⇌\\ k_{r_{i}} \end{array}D_{i}$$

We measure $L_{i}$ as the concentration of ligand in a volume V and $R_{i}$ and $D_{i}$ as the absolute numbers of receptors or complexes on the cell surface. The differential equations describing their dynamics are then as below:

(Equation 16) $$\frac{dL_{i}}{dt}=\frac{1}{V}\left(-k_{f_{i}}L_{i}R_{i}+k_{r_{i}}D_{i}\right)$$

(Equation 17) $$\frac{dR_{i}}{dt}=-k_{f_{i}}L_{i}R_{i}+k_{r_{i}}D_{i}$$

(Equation 18) $$\frac{dD_{i}}{dt}=k_{f_{i}}L_{i}R_{i}-k_{r_{i}}D_{i}$$

We again assume that the volume for ligands is large, such that there are significantly more ligand molecules than receptors. This assumption holds for our experimental setting, where ligands are dissolved in an excess of media. Ligand concentrations thus remain constant. We enforce conservation of mass for the receptor subunits. Finally, we define affinities $K_{i}\equiv k_{f_{i}}/k_{r_{i}}$ and activities $e_{i}\equiv \varepsilon_{i}/\gamma$ as before. At steady state, we have the following equations to describe the behavior of a model with n ligands and receptors:

(Equation 19) $$L_{i}^{0}=L_{i}$$

(Equation 20) $$R_{i}^{0}=R_{i}+D_{i}$$

(Equation 21) $$D_{i}=K_{i}L_{i}R_{i}$$

(Equation 22) $$S=\sum_{i=1}^{n}e_{i}D_{i}$$

The steady-state solutions for $D_{i}$ can then be derived as

(Equation 23) $$D_{i}=K_{i}L_{i}^{0}\left(R_{i}^{0}-D_{i}\right)=\frac{K_{i}L_{i}^{0}R_{i}^{0}}{1+K_{i}L_{i}^{0}}$$

##### One-step vs. two-step model

We have previously considered a mathematical model that describes the promiscuous architecture of the BMP pathway, which considers formation of the heterotrimeric complexes $T_{ijk}$ in a two-step process (Antebi et al., 2017). Briefly, ligand $L_{i}$ and receptor $A_{j}$ form an intermediate dimer $D_{ij}$, which then binds to receptor $B_{k}$ to form trimer $T_{ijk}$. Again, we assume that the reactions are reversible and follow first-order kinetics, with forward and reverse reaction rates $k_{f_{ij}}^{D}$ and $k_{r_{ij}}^{D}$ for formation of the dimers and $k_{f_{ijk}}^{T}$ and $k_{r_{ijk}}^{T}$ for formation of the trimers. Defining $K_{ij}^{D}\equiv k_{f_{ij}}^{D}/k_{r_{ij}}^{D}$ and $K_{ijk}^{T}\equiv k_{f_{ijk}}^{T}/k_{r_{ijk}}^{T}$, the steady-state solutions for $T_{ijk}$ in the two-step model, analogous to Equation 14 in the one-step model, are as follows:

(Equation 24) $$T_{ijk}=K_{ijk}^{T}K_{ijk}^{D}L_{i}\left(\frac{A_{j}^{0}-\sum_{i^{\prime }=1}^{n_{L}}\sum_{k^{\prime }=1}^{n_{B}}T_{i^{\prime }jk^{\prime }}}{1+\sum_{i^{\prime }=1}^{n_{L}}K_{i^{\prime }j}^{D}L_{i^{\prime }}}\right)\left(B_{k}^{0}-\sum_{i^{\prime }=1}^{n_{L}}\sum_{j^{\prime }=1}^{n_{A}}T_{i^{\prime }j^{\prime }k}\right)$$

Comparing Equations 14 and 24, the steady-state solutions for $T_{ijk}$ in the two-step model can be mapped to the one-step model under the following parameter choice:

(Equation 25) $$K_{ijk}=\frac{K_{ijk}^{T}K_{ij}^{D}}{1+\sum_{i^{\prime }=1}^{n_{L}}K_{i^{\prime }j}^{D}L_{i^{\prime }}}$$

Since S is defined by the values of $T_{ijk}$ and is given by Equation 11 in both the one-step and two-step models, the steady-state behavior of the two-step model with any set of parameters can also be represented in the one-step model. However, the number of parameters is reduced from $N_{p}^{two-step}=n_{A}+n_{B}+n_{L}n_{A}+2n_{L}n_{A}n_{B}$ to $N_{p}^{one-step}=n_{A}+n_{B}+2n_{L}n_{A}n_{B}$. Thus, the one-step model enables us to simplify the system while preserving all possible behaviors of $T_{ijk}$ and S at steady state.

##### Trimeric vs. hexameric model

We have developed a simplified model in which a ligand binds to type I and type II receptor subunits to form a trimeric signaling complex. However, the BMP signaling pathway is known to involve hexameric signaling complexes, where a dimeric ligand interacts with two type I and two type II receptors. This model captures reactions of the following form:

(Equation 26) $$L_{i}^{1}+L_{j}^{2}+A_{k}^{1}+A_{l}^{2}+B_{m}^{1}+B_{n}^{2}\begin{array}{c} k_{f_{ijklmn}}\\ ⇌\\ k_{r_{ijklmn}} \end{array}H_{ijklmn}$$

This model can essentially be reduced to a trimeric model by setting reaction rates to 0 for any reaction with i≠j, k≠l, or m≠n. As such, responses in the trimeric model represent a subset of the functions that could be possible in the hexameric model.

#### Optimization of orthogonal addressing schemes

Given a target orthogonal addressing scheme of N channels, we optimized for parameters that would yield matching responses. Specifically, we used constrained least-squares optimization for $n_{L}n_{A}n_{B}$ affinity parameters $K_{ijk}$, $n_{L}n_{A}n_{B}$ activity parameters $e_{ijk}$, and $N\left(n_{A}+n_{B}\right)$ receptor expression levels. We bounded affinity and efficiency parameters in [0,1] and receptor levels in $\left[0,\infty \right)$. We sought to minimize the residuals between the target responses and the simulated responses at the N ligand words of interest. Since the simulated responses have arbitrary units, we normalized all responses for a given parameter set. In particular, we normalized by the maximum value in any cell type over the full ligand titration, not only the ligand words of interest. This normalization ensures that all cell types share a relatively similar level of activation and that the activation in the orthogonal channels is distinguishable from activation by other ligand combinations.

As this optimization procedure is not guaranteed to converge to a global minimum, we optimized repeatedly with different initial conditions. Biochemical parameters were chosen in a uniform random distribution over [0,1], and receptor levels were initialized to 1. To evaluate the potential capacity of promiscuous ligand-receptor systems for orthogonal addressing, we sought to optimize progressively higher bandwidths without requiring any particular scheme. For N channels, we randomly selected N ligand words as orthogonally activating inputs and sought to optimize parameters as described. We iterated this process with randomly chosen ligand words until parameters had been identified to generate N channels successfully. Once this criterion was met, we then proceeded to optimize N+1 channels, up to the limit derived from the number of possible ligand words. We performed at least an average of 500 optimizations per bandwidth.

#### Enumeration of orthogonal addressing schemes

To analyze the possibility for orthogonal addressing, we used a discretized ligand concentration space to enable a comprehensive screening. We reasoned that we could systematically test for all possible orthogonal addressing schemes by selecting a subset of the possible ligand combinations to be orthogonally activating and defining a set of targeted response functions accordingly. For a set of N chosen ligand words, we enumerated N targeted response functions, where each ligand word activates exactly one cell type and, conversely, each cell type is activated by exactly one ligand word. Having discretized ligand concentrations to three levels, there are $3^{2}=9$ possible ligand words. Since the combination with all ligands at the lowest level is assumed to yield negligible activation in any cell type, there can be one to eight possible communication channels.

For each possible number of channels N, we took all possible subsets and sought to achieve these addressing schemes. There are $\left(\begin{array}{l} 8\\ N \end{array}\right)$ possible addressing schemes for a given bandwidth and $2^{8}=256$ possible addressing schemes overall. However, addressing schemes that are identical under changes in ligand labels were removed, leaving 144 total possibilities. We systematically tested for the ability to achieve each addressing scheme by performing a search over all schemes. At each trial, we chose which addressing scheme to optimize based on both the lowest error E achieved and the number of trials T attempted. Specifically, we optimized for the addressing scheme with maximal value of $E/T^{2}$ and repeated this process for at least an average of 50 optimizations per scheme to ensure that all schemes would be tested adequately. This strategy, optimizing each addressing scheme rather than randomly selecting schemes of a given bandwidth, provided a complementary approach for analyzing addressing capability.

#### Distinguishability of channels

We optimized parameters for each addressing scheme based on the squared error between the targeted and simulated responses at each ligand word of interest. However, this error does not necessarily guarantee specificity of addressing, where a given ligand combination should activate only a single cell type and not the others. To quantify the performance of each system, we analyzed the distributions of on-target and off-target activation levels. We defined the distinguishability as the fold difference between the minimum on-target and maximum off-target activities, which measures the ability to differentiate between specific and nonspecific signals in the worst case.

#### Orthogonal addressing in one-to-one model

##### Lower bound for distinguishability

For a two-component one-to-one system, we can readily use Equations 22 and 23 to calculate the steady-state signal for any set of initial ligand and receptor concentrations under a given set of biochemical parameters:

(Equation 27) $$S=\frac{K_{1}e_{1}L_{1}^{0}R_{1}^{0}}{1+K_{1}L_{1}^{0}}+\frac{K_{2}e_{2}L_{2}^{0}R_{2}^{0}}{1+K_{2}L_{2}^{0}}$$

Suppose that $K_{1}=K_{2}$ and $K_{i}\gg L_{i}^{0}$ for any i. Define $Q_{i}=e_{i}R_{i}^{0}$. In this regime, the signal (subject to an arbitrary normalization factor) is approximately as below:

(Equation 28) $$S\approx L_{1}^{0}Q_{i}+L_{2}^{0}Q_{2}$$

To obtain an addressing system of N orthogonal channels, we must define N ligand words and N cell types. Let $W_{i}$ denote the ith word, or a length-2 vector representing a ligand expression profile as $W_{i}={\left(L_{1}^{0},L_{2}^{0}\right)}^{\left(i\right)}$. Likewise, let $C_{j}^{'}$ represent the jth cell type, or a length-2 vector representing a receptor expression profile as $C_{j}^{'}={\left(R_{1}^{0},R_{2}^{0}\right)}^{\left(j\right)}$. For mathematical convenience, we can instead consider $C_{j}={\left(Q_{1},Q_{2}\right)}^{\left(j\right)}$, which encompasses receptor expression as well as activity. Letting $S_{ij}$ denote the steady-state signal for the ith ligand word and the jth cell type, this value is simply $S_{ij}=W_{i}\cdot C_{j}$. As such, we wish to maximize $W_{i}\cdot C_{i}$ and minimize $W_{i}\cdot C_{j}$ (for i≠j) in order to maximize distinguishability. Since the dot product between any two vectors a and b with an angle θ between them is given by a·b=‖a‖‖b‖cosθ, we should choose $W_{i}$ and $C_{i}$ to be directly proportional to one another (i.e., θ=0, thus maximizing cosθ), and we should choose the different $W_{i}$ to be equidistantly spaced across the “unit arc” or first quadrant of the unit circle (thus maximizing θ and minimizing cosθ). Specifically, we can define $W_{i}=C_{i}=\left(\cos\;\theta_{i},\sin\;\theta_{i}\right)$ for $\theta_{i}=\frac{i-1}{N-1}\cdot \frac{\pi }{2}$ radians. Each on-target signal will then simply be $S_{ii}=1$, and each off-target signal will be $\cos\left(\frac{\pi }{2\left(N-1\right)}\right)$. Thus, we can guarantee implementation of N orthogonal channels with a distinguishability of at least

(Equation 29) $$D=\frac{1}{\cos\left(\frac{\pi }{2\left(N-1\right)}\right)}$$

##### Optimization

The above analysis guarantees implementation of arbitrarily many orthogonal channels with distinguishability greater than 1. However, it makes several assumptions. Therefore, we also sought to identify parameters that could improve upon the class of solutions derived above in the absence of such simplifications. We used an optimization approach, optimizing for distinguishability and searching over a single set of affinity and activity parameters as well as N sets of ligand and receptor expression values. For the same bandwidths as considered for the promiscuous system, we performed 100 optimization trials each. In all cases, the resulting distinguishability values approached but did not exceed the theoretical value. Thus, our theoretical solutions, while not proven to be optimal, are able to outperform computationally optimized parameters.

#### Analysis of robustness

Biological systems are subject to noise. In particular, cellular systems show both extrinsic noise, or correlated changes such as during cell growth or changes in expression machinery, and intrinsic noise, or independent stochastic variation in each element. To assess whether the optimized parameters are robust to noise in receptor expression levels, we evaluated whether on-target and off-target signals could be correctly distinguished across many random perturbations, using the receiver operating characteristic (ROC). In particular, we computed the area under the ROC curve (AUC), which represents the probability of successfully classifying on-target from off-target activations. We considered both purely extrinsic and purely intrinsic noise. For a given coefficient of variation (CV) ν (here, ν=0.5), we simulated extrinsic noise by generating a scale factor from a gamma distribution with shape parameter $1/\nu^{2}$ and scale parameter $\nu^{2}$ (giving a mean of 1 and a variance of $\nu^{2}$) and multiplied all receptor levels by this scale factor. For intrinsic noise, we instead drew scale factors i.i.d. for each receptor. With each form of noise, we generated 100 random perturbations, simulated the resulting activity levels, and computed the corresponding ROC and AUC (Figures 3C and 3G). We also plotted the distinguishability values for these perturbations (Figure S3B).

#### Optimization of subset addressing repertoires

We next considered more general addressing systems, targeting activation not just of individual cell types but also of groups of cell types. Specifically, an addressing repertoire encompasses all subsets of cell types that can be co-activated by any ligand word across a complete titration of ligand concentrations. For instance, titrating two ligand variants with three concentrations yields $3^{2}=9$ ligand words, each of which activates some subset of the cell types considered. Every distinct group of cell types constitutes an achievable channel. The set of channels resulting from the 9 ligand words considered constitutes the addressing repertoire for that set of parameters.

To characterize the specificity of addressing different subsets of cell types, we generalized the distinguishability metric defined above. Each ligand word activates a particular subset of cell types; the corresponding response(s) of the cell type(s) would represent on-target signaling, while the response(s) of any other cell type(s) would represent off-target signaling. Therefore, as in the case of orthogonal addressing, distinguishability can be calculated as the fold difference between the minimum on-target activity and the maximum off-target activity.

Similar to a specific number of orthogonal channels, a given addressing repertoire can potentially be implemented in many ways. In other words, many different sets of responses can generate the same addressing repertoire. Unlike the orthogonal case, however, the responses for all ligand combinations are relevant. Thus, enumerating the ways to achieve a given addressing repertoire requires considering any possible response for every cell type.

To generalize our optimization approach to analyze addressing repertoires, we first set out to define what sets of responses could yield a given repertoire. Therefore, we started by enumerating all possible binary response matrices for a single cell type. The number of possible responses then reduces to the number of ways to choose “on” signals. Assuming that cells are always inactive for the ligand combination where both ligands are present at low levels and ignoring the case where the cell is entirely nonresponsive, there are $2^{8}-1=255$ possible responses.

By considering all combinations of three responses from this set, we were able to map all addressing repertoires to the potential sets of three responses. Due to the large number of possibilities for a given repertoire, we sought to prioritize sets of responses that were more likely to be achievable. Therefore, we individually optimized each of the 255 responses and quantified the quality of each response using the sum of squared distances to the target, after normalizing the simulated response to have a maximum response of 1 (data not shown). We ranked sets of three responses based on the sum of the scores of each response individually. Since parameter sets were individually optimized, a response that can be achieved with high quality independently may not be possible in the same biochemical parameter regime as another; however, this scoring should reduce consideration of responses that are challenging to optimize individually, let alone together with others.

Having selected candidate sets of responses, we could then perform least-squares optimization as done previously. We also complemented this optimization approach by reasoning that any given set of responses matches some addressing repertoire, depending only on how the threshold between “inactive” and “active” pathway response is defined. Therefore, we simulated a random set of responses, chose the threshold that yielded the greatest distinguishability between the lowest on-target and highest off-target responses, and associated those parameters with the resulting addressing repertoire. We iteratively optimized for this distinguishability, stopping if the resulting addressing repertoire was one for which a valid parameter set had not yet been identified.

#### Enumeration of subset addressing repertoires

We focused on analyzing addressing repertoires for three cell types, denoted A, B, and C. With three cell types, there are eight possible channels: one with no cell types activated, three with a single cell type activated, three with two cell types activated, and one with all cell types activated. Since the channel with no cell types activated is always achieved in the absence of any ligand, we neglect this from further consideration. Each of the remaining seven channels may or may not be present, for a total of up to $2^{7}=128$ addressing repertoires. We discard repertoires that are redundant with respect to relabeling of ligands as well as repertoires in which two cell types are indistinguishable by any ligand combination (such that the addressing repertoire could be mapped to a repertoire for two cell types). As discussed in more detail below, these simplifications leave us with 32 addressing repertoires of three cell types, corresponding to those shown in Figure 4C.

We first seek to eliminate repertoires that are invariant with respect to relabeling of ligands. The subset with all three cell types activated, or triple, does not change when ligands are relabeled; however, singles or doubles may. For example, the addressing repertoire consisting of “A” and “BC” is equivalent to that comprising “B” and “AC,” simply by swapping the labels of cell types A and B. As such, we consider the unique ways to include singles or doubles. Consider each single with its complementary double (for example, “A” with “BC”). There are $2^{2}=4$ possible ways to include this pair in an addressing repertoire: both absent, only single present, only double present, and both present. The three pairs can then encompass three distinct choices of these four possibilities (4 combinations), two distinct choices (4·3=12 combinations), or the same choice (4 possibilities). There are then 4+12+4=20 ways to choose combinations of singles or doubles, and the triple may be either present or absent. Thus, considering ligand relabeling reduces the total number of addressing repertoires to consider to 40.

Note, however, that some of these repertoires may only represent two distinct cell types, rather than three. For example, the addressing repertoire with channels “A” and “BC” indicates that cell types B and C are indistinguishable across all ligand combinations and are therefore functionally equivalent. Let B and C be indistinguishable, without loss of generality. The only possible channels are then “A,” “BC,” and “ABC.” Thus, the $2^{3}=8$ repertoires that only contain these channels can be reduced to two distinct cell types and are therefore omitted from our analysis of addressing three cell types. This correction yields our final set of 32 addressing repertoires.

#### Subset addressing in one-to-one model

To understand how subset addressing in a promiscuous pathway compares with that in a one-to-one architecture, we note that all responses in a one-to-one pathway must be monotonic, meaning that responses never decrease with added ligand. As such, a given cell's response is maximal when exposed to the ligand combination where all ligands are present at highest concentration. Therefore, every cell type will be active in response to this ligand combination. (Otherwise, there would be no response across the entire ligand titration, and there would be no addressing.) Consequently, the subset “ABC” will always be addressable in the one-to-one architecture. Conversely, any repertoire where “ABC” is absent cannot be achieved in the one-to-one architecture.

#### Addressing of cell lines

##### Receptor knockdown and ectopic expression

To analyze the potential for addressing in living cells, we engineered cell lines with differing receptor profiles. Using an NMuMG reporter line, individual BMP receptors were knocked down (KD) or overexpressed (OX), as described in (Klumpe et al., 2022). Briefly, receptor knockdown lines were engineered by transducing lentiviral particles containing constructs for constitutive shRNA expression reported by mCherry with a puromycin resistance gene (SMARTvector, Dharmacon). ACVR1 KD cells were a clonal population selected by limiting dilution of cells transduced with a pool of three shRNAs, while BMPR2 KD cells were a polyclonal population generated by a single shRNA. Cells were selected 48 hours after transduction and continuously maintained in 3 μg/mL puromycin. For ectopic expression of BMP receptors, a construct for constitutive expression of mouse receptor cDNA reported by mTurquoise and co-expressed with a geneticin resistance gene was integrated by PiggyBac integration (System Biosciences) using previously reported plasmids (Antebi et al., 2017). ACVRL1 OX cells were selected and maintained in 500 μg/mL geneticin.

##### BMP response and flow cytometry

Responses of cell lines to BMP ligands were quantified by flow cytometry as described in (Klumpe et al., 2022). Cells were plated at 20-30% confluency in 96-well plates and cultured under standard conditions for 12 hours. Media was then replaced, and ligands were added at specified concentrations. 24 hours after ligand addition, cells were prepared for flow cytometry by washing with PBS and lifting from the plate using either trypsin (NMuMG) or Accutase (mESC) for 5 minutes at 37°C. Protease activity was quenched by resuspending the cells in HBSS with 2.5 mg/mL bovine serum albumin (BSA). Cells were then filtered with a 40 μm mesh and analyzed by flow cytometry (MACSQuant VYB, Miltenyi Biotec; CytoFLEX, Beckman Coulter). Recombinant BMP ligands were acquired from R&D Systems (BMP2, catalog #355-BM; BMP9, catalog #5566-BP; BMP10, catalog #6038-BP).

#### Computation of mutual information

We use mutual information between ligand words and activation patterns across a library of cell types to quantify the combinatorial addressing power of the ligand-receptor system. Mutual information was initially developed to quantify the capacity of a noisy channel to transmit information, or the extent to which distinct input messages can be resolved by the receiver after passing through the channel. Here, we view the ligand words as input messages and the resulting activation pattern across cell types as the received message. Then, the communication system's capacity is determined by the biochemical constants $K_{ijk}$ and $e_{ijk}$.

One important benefit of using an information theoretic framework is that we do not need to assume a particular set of ligand words and cell types and then optimize over them. Instead, we can use extensive libraries of input ligand words and cell types; mutual information will reflect the best subset of each with no penalty (or benefit) for redundancies. Thus, in our framework, mutual information reflects a property of the biochemical constants K and e alone; ligand inputs and cell types are implicitly assumed to be optimally chosen. (In information theoretic language, we do not need to know optimal error-correcting codes to compute the capacity of a channel.)

Let W represent a library of $n_{LW}$ ligand words, where the ith input $W_{i}$ is a vector of $n_{L}$ ligand concentrations. Given a library of $n_{CT}$ cell types, let S(W) represent the resulting activation profiles of these cell types, or a set of $n_{LW}\times n_{CT}$ responses. Earlier sections have presented a way to compute S(W) deterministically by solving quadratic equations. Here, we assume that the activation is probabilistic due to a Gaussian error bar of size σ around the deterministic solution $S_{\mathrm{determ}}\left(W\right)$. The standard deviation σ can represent molecular fluctuations upstream of SMAD (e.g., in receptor levels or activity) that result in fluctuations of SMAD phosphorylation. Thus, the distribution of signaling activities can be represented as follows:

(Equation 30) $$P\left(S|W\right)=\text{Normal}\left(S_{\text{determ}}\left(W\right),\sigma^{2}\right)$$

We compute mutual information MI(S,W) using the formula below:

(Equation 31) MI(S,W)=H(S)−H(S|W)

The second term can be expressed as follows:

(Equation 32) $$H\left(S|W\right)=\sum_{i=1}^{n_{LW}}p\left(W_{i}\right)H\left(S|W=W_{i}\right)$$

Each $H\left(S|W=W_{i}\right)$ is the entropy of a $n_{CT}$-dimensional Gaussian with covariance matrix $\Sigma =\sigma^{2}I$, where I is the identity matrix of size $n_{CT}$. This entropy (in bits) is

(Equation 33) $$\begin{array}{l} H\left(S|W=W_{i}\right)=\frac{1}{2}\mathrm{lg}\left[\det\left(2\pi e\Sigma \right)\right]\\ =\frac{1}{2}\mathrm{lg}\left[{\left(2\pi e\sigma^{2}\right)}^{n_{CT}}\right]\\ =\frac{n_{CT}}{2}\mathrm{lg}\left[2\pi e\sigma^{2}\right] \end{array}$$

Assuming input probabilities are uniformly distributed, or $p\left(W_{i}\right)=\frac{1}{n_{LW}}$, this conditional entropy is simply given by

(Equation 34) $$H\left(S|W\right)=\frac{n_{CT}}{2}\mathrm{lg}\left[2\pi e\sigma^{2}\right]$$

Thus, this term is constant regardless of choice of biochemical parameters.

The entropy H(S) in Equation 31 is the entropy of P(S), which is a sum of Gaussians, one at each of the activation patterns corresponding to each ligand input $W_{i}$. This entropy is a measure of the distinguishability of activation patterns $S\left(W_{i}\right)$ for different inputs $W_{i}$; the entropy will be small if the Gaussians are overlapping and large otherwise. Intuitively, this entropy is a measure of how well separated the activation patterns for different ligand inputs are.

The problem of determining the entropy of a normalized sum of Gaussians (i.e., a Gaussian mixture) in high dimensions is complex; however, simple analytic approximations have been developed in a recent advance (Kolchinsky and Tracey, 2017). We use the approximation to the kernel density estimator presented therein for a sum of $n_{LW}$ Gaussians $p\left(x\right)=\frac{1}{n_{LW}}\overset{n_{LW}}{\underset{i=1}{\sum }}p_{i}\left(x\right)$ in $n_{CT}$ dimensions:

(Equation 35) $$H_{KL}\left(p\left(x\right)\right)=\frac{n_{CT}}{2}-\sum_{i=1}^{n_{LW}}m_{i}\;\ln\left[\sum_{j=1}^{n_{LW}}m_{j}p_{j}\left(\mu_{i}\right)\right]$$

Here, $p_{j}$ is the jth Gaussian component (normalized to 1, individually), $\mu_{i}$ the mean of the ith component, and $m_{i}$ the mixture weight of the ith component. In this case, we assume uniform mixture weights, or $m_{i}=\frac{1}{n_{LW}}$ for all i. Further, $p_{j}\left(\mu_{i}\right)$ is

(Equation 36) $$p_{j}\left(\mu_{i}\right)=\frac{1}{\sqrt{{\left(2\pi \right)}^{n_{CT}}\det\Sigma }}e^{-\frac{1}{2}{\left(\mu_{i}-\mu_{j}\right)}^{T}\Sigma^{-1}\left(\mu_{i}-\mu_{j}\right)}$$

Thus, the mutual information can be evaluated by simply evaluating each Gaussian at the mean of all other Gaussian components, or by using the matrix $D_{ij}$ of distances between activation patterns $S_{\mathrm{determ}}\left(W_{i}\right)$ for different ligand inputs $W_{i}$:

(Equation 37) $$D_{ij}=\|S_{\text{determ}}\left(W_{i}\right)-S_{\text{determ}}\left(W_{j}\right){\|}^{2}$$

We can therefore simplify $p_{j}\left(\mu_{i}\right)$ to

(Equation 38) $$p_{j}\left(\mu_{i}\right)=\frac{1}{\sqrt{{\left(2\pi \sigma^{2}\right)}^{n_{CT}}}}\;e^{-\frac{D_{ij}}{2\sigma^{2}}}$$

Substituting into the approximation, we find an entropy (in nats) of

(Equation 39) $$\begin{array}{l} H_{KL}\left(P\left(S\right)\right)=\frac{n_{CT}}{2}-\sum_{i=1}^{n_{LW}}\frac{1}{n_{LW}}\ln\left[\sum_{j=1}^{n_{LW}}\frac{1}{n_{LW}}\cdot \frac{1}{\sqrt{{\left(2\pi \sigma^{2}\right)}^{n_{CT}}}}\;e^{-\frac{D_{ij}}{2\sigma^{2}}}\;\right]\\ =\frac{n_{CT}}{2}-\frac{1}{n_{LW}}\sum_{i=1}^{n_{LW}}\ln\left[\frac{1}{n_{LW}}\cdot \frac{1}{\sqrt{{\left(2\pi \sigma^{2}\right)}^{n_{CT}}}}\sum_{j=1}^{n_{LW}}e^{-\frac{D_{ij}}{2\sigma^{2}}}\;\right]\\ =\frac{n_{CT}}{2}-\frac{1}{n_{LW}}\sum_{i=1}^{n_{LW}}\left(-\ln\left[n_{LW}\right]-\frac{n_{CT}}{2}\ln\left[2\pi \sigma^{2}\right]+\ln\left[\sum_{j=1}^{n_{LW}}e^{-\frac{D_{ij}}{2\sigma^{2}}}\right]\right)\\ =\frac{n_{CT}}{2}+\ln\left[n_{LW}\right]+\frac{n_{CT}}{2}\ln\left[2\pi \sigma^{2}\right]-\frac{1}{n_{LW}}\sum_{i=1}^{n_{LW}}\left(\ln\left[\sum_{j=1}^{n_{LW}}e^{-\frac{D_{ij}}{2\sigma^{2}}}\right]\right)\\ =\ln\left[n_{LW}\right]+\frac{n_{CT}}{2}\ln\left[2\pi e\sigma^{2}\right]-\frac{1}{n_{LW}}\sum_{i=1}^{n_{LW}}\left(\ln\left[\sum_{j=1}^{n_{LW}}e^{-\frac{D_{ij}}{2\sigma^{2}}}\right]\right) \end{array}$$

We can convert this expression to bits by multiplying by lg2 and then combine with Equation 34 to estimate mutual information. We note that these derivations omit a correction factor $-n_{CT}\mathrm{lg}\text{Δ}S$ arising from binning with bin width ΔS to make the entropy of a continuous distribution well defined; however, as the same correction applies to both H(S) and H(S|W), these terms cancel out. Our estimator of mutual information is therefore

(Equation 40) $$\begin{array}{l} MI\left(S,W\right)=H\left(S\right)-H\left(S|W\right)\\ =\mathrm{lg}\;e\left(\ln\left[n_{LW}\right]+\frac{n_{CT}}{2}\ln\left[2\pi e\sigma^{2}\right]-\frac{1}{n_{LW}}\sum_{i=1}^{n_{LW}}\left(\ln\left[\sum_{j=1}^{n_{LW}}e^{-\frac{D_{ij}}{2\sigma^{2}}}\right]\right)\right)-\frac{n_{CT}}{2}\mathrm{lg}\left[2\pi e\sigma^{2}\right]\\ =\mathrm{lg}\left[n_{LW}\right]-\frac{1}{n_{LW}}\sum_{i=1}^{n_{LW}}\mathrm{lg}\left[\sum_{j=1}^{n_{LW}}e^{-\frac{D_{ij}}{2\sigma^{2}}}\right] \end{array}$$

We use this expression to estimate mutual information in this paper. From the form, it is clear that mutual information rewards large values of $D_{ij}$, or distinct activation patterns for different ligand inputs.

The mutual information framework above can be naturally extended to scenarios not considered here. For example, not all ligand inputs might be equally likely or of equal physiological significance. In this case, the map of ligand inputs to activation profiles (i.e., the coding scheme) can separate the activation patterns of more important ligand words at the expense of more similar activation patterns for less important words. The mutual information framework can account for such weighting of different inputs easily through unequal $p\left(W_{i}\right)$ above.

Finally, note that mutual information naturally rewards robustness, since mutual information is higher when each activation pattern is realized over equally sized regions of input space. For example, if an output $S_{1}$ is only obtained for a sliver of ligand input space while another output pattern $S_{2}$ is realized over the rest of input space, mutual information will be lower than if both outputs are realized over half of input space.

#### Analysis of mutual information

##### Libraries of ligand words and cell types

To provide the broadest information theoretic characterization, we first constructed comprehensive libraries of ligand words, or input messages, and cell types, or receptor expression profiles. Each ligand can independently take on three distinct concentrations sampled logarithmically over three orders of magnitude, or $10^{0}=1$, $10^{1.5}\approx 32$, and $10^{3}=1000$. This library of $3^{2}=9$ words is a representative sampling of all possible ligand inputs. Similarly, we constructed a library of cell types by varying each receptor level independently over two distinct concentration levels [1,100]. For a system with two type I receptor variants and two type II receptor variants, this library comprises $2^{2+2}=16$ possible cell types.

##### Systematic sampling of biochemical parameters

In our model, a promiscuous ligand-receptor system is defined by its interaction affinities K and signaling activities e. To comprehensively sample all possible biochemical parameters, each parameter was allowed to be either of [0.1,1], giving a total of $2^{16}=65,536$ qualitatively distinct parameter sets. We then evaluated mutual information between the ligand words and the corresponding cell type activation profiles for each possible choice of biochemical parameters (K,e). The resulting data characterize the combinatorial addressing power across a comprehensive set of promiscuous ligand-receptor systems.

##### Random sampling of biochemical parameters

To ensure that the grid-based sampling procedure did not introduce any artifacts, we also repeated this analysis for an identical number of randomly generated parameter sets. Specifically, we chose each value independently and randomly with a log-uniform distribution over $\left[10^{-1.5},1\right]$. The resulting distribution of mutual information values is shown in Figure S6A.

##### Choice of variance

Computing mutual information requires choosing the Gaussian fluctuation or variance $\sigma^{2}$ for activation levels. For all results here, we choose $\sigma^{2}=0.5$, based on testing a range of values. Very large or small choices of σ lead to the same value of mutual information for all biochemical parameters, either low or high, respectively. Intermediate choices of σ discriminate between different (K,e). While the precise value of mutual information depends on σ, different choices of σ do not qualitatively change the relative ordering of biochemical parameter sets.

#### Optimization of mutual information

These sampling procedures enable us to comprehensively analyze mutual information across parameter space. However, they are likely to miss extremes of mutual information. Therefore, we chose the 16 parameter sets from the systematic grid-based sampling with the highest starting mutual information and further refined (K,e) to maximize mutual information, using least-squares optimization.

#### Addressability of ligand words

To determine which ligand words in the library activate distinct combinations of cell types, we define the overall addressability of a set of ligand words by evaluating all pairs. To compare a pair of ligand words, we compute the ratio of activation levels for each cell type and take the separation r as the largest such fold change (inverted if needed, such that r≥1). If two ligand words have a separation of 5, then at least one cell type's activation is different by a factor of at least 5 in the two conditions. We extend this pairwise separation to a set of N ligand words by forming a N×N addressability matrix, where element (i,j) corresponds to the pairwise separation of ligand words i and j. This matrix has 1s along the diagonal. We define the smallest off-diagonal value, which represents the minimum pairwise separation between different ligand words, to be the overall addressability of that set of N ligand words.

#### Analysis of archetypal responses

##### Response classes

We next analyzed the responses generated by the full library of cell types for high-performing and low-performing parameter sets. As expected, parameter sets giving rise to low mutual information showed relatively little diversity in responses (Figure 6D); parameter sets which generated high mutual information showed distinct activation patterns among cell types (Figure 6E). Furthermore, these response types appeared qualitatively different and were similar to experimentally observed patterns reported previously (Antebi et al., 2017). We therefore further analyzed the presence of these archetypal responses across parameter sets.

Examples of these archetypes were generated by simulating responses to parameters reflecting our understanding of the underlying design principles (Figure S1). These parameters are not specifically tuned, with all affinity and activity values set to either 0.1 or 1 and all receptor levels fixed at 10^-1.5^. Thus, they reflect qualitative differences rather than finely tuned quantitative ones. Briefly, ratiometric responses feature reduction of activity of one ligand by the second, such that the overall response approximates the ratio of the two concentrations. Competitive inhibition, where the “denominator” competes for receptors needed to generate signaling activity but produces inactive complexes, can produce such responses (Figure S1A). Additive responses approximate the sum of the two ligand concentrations, as the ligands increase pathway activity either alone or together, and are readily generated when both ligands activate receptors similarly (Figure S1B). Imbalance detection responses, where cells respond maximally to imbalances in the levels of the two ligands, can arise if, for instance, competition between two ligands favors complexes with low signaling activity (Figure S1C). Conversely, balance detection responses, where cells respond maximally when both ligands are present at a specific ratio, can be generated when ligand binding favors formation of high-activity signaling complexes (Figure S1D).

##### Phenotypical parameters

We characterized the spectrum of responses as described previously (Antebi et al., 2017). Briefly, we use the relative ligand strength (RLS), which represents the ratio of activation produced by the weaker ligand to that produced by the stronger ligand, and the ligand interference coefficient (LIC), which measures the degree to which two ligands positively or negatively synergize. We computed these values for each of the 16 responses of the cell type library for each set of biochemical parameters and determined what response classes each fell into, adapting previously described criteria (Antebi et al., 2017). Ratiometric responses were defined by RLS<0.2, additive responses by RLS>0.8 and |LIC|<0.05, imbalance responses by RLS>0.8 and LIC<−0.1, and balance responses by RLS>0.8 and LIC>0.1. Responses outside these ranges were considered to be intermediate variants and were not classified as a particular archetype.

##### Relationship with mutual information

Having identified the response classes represented for each set of biochemical parameters, we plotted the distribution of mutual information values associated with a given number of response classes (Figure 6F).

#### Analysis of parameter correlations

Based on observations from parameter sets with high mutual information, we computed two correlation measures for the biochemical parameters. Since each parameter could only take on two values, we transformed them to -1 and 1. In particular, we defined $K_{ijk}^{'}=-1$ if $K_{ijk}=0.1$ (low) and $K_{ijk}^{'}=1$ if $K_{ijk}=1$ (high), with $e_{ijk}^{'}$ defined analogously. (Equivalently, we defined $K_{ijk}^{'}=1+2\log_{10}\;K_{ijk}$.) We computed $H_{Ke}=\sum_{i,j,k}K_{ijk}^{\prime }e_{ijk}^{\prime }$ to measure correlation between binding and signaling efficiency for each signaling complex. We also computed $H_{ee}=\sum_{j,k}e_{1jk}^{\prime }e_{2jk}^{\prime }$ to measure the correlation between activities of the two signaling complexes with the same receptor dimer but different ligands.

We calculated each correlation metric for every parameter set. To investigate the relationship with mutual information, we sorted parameter sets by mutual information, binned them (with a bin size of 800), and computed the average correlation and mutual information in each bin (Figures 6G and 6H).

#### Evolutionary algorithm as a generative model

While the observed anticorrelations of K and e appear to be predictive of mutual information, it is not clear if these relationships are sufficient to fully describe the criteria for high addressing power and can thus serve as a design principle. Therefore, we developed a generative algorithm that systematically evolves a given set of parameters (K,e) according to these principles and asked whether favoring anticorrelations yields higher addressing power.

We first formed a fitness function $F\left(K,e\right)=-\left(H_{Ke}+H_{ee}\right)$, where $H_{Ke}$ and $H_{ee}$ are as above. Intuitively, a choice of (K,e) that has strong affinity-activity or activity-activity anticorrelations would have high fitness. Our algorithm is then a simple “evolutionary” algorithm that performs noisy gradient ascent in this fitness landscape. Starting with a given (K,e), each iteration involves choosing a random element of (K,e) and proposing a flip (changing it to high if currently low or vice versa). We then compute the resulting change in fitness ΔF. If such a flip increases fitness (ΔF>0), we immediately implement it. If the proposed flip decreases fitness (ΔF<0), we accept it with a probability $e^{s\Delta F}$, where s represents the selection pressure. Such moves towards lower fitness allow dynamics to escape local fitness maxima; the frequency of such moves towards lower fitness is controlled by the selection pressure s (or, equivalently, temperature in Monte Carlo algorithms). We repeat this process over many iterations and track the addressing power of the resulting (K,e) configuration.

We ran our algorithm on 2,000 randomly initialized choices of (K,e). For each initialization, we performed 200 iterations of the evolutionary algorithm and quantified the addressing capacity of the final (K,e) using mutual information. We then visualized the resulting distribution of mutual information values (Figure 6K). With s=0 (i.e., no selection for particular parameter relationships), there is a wide histogram equivalent to random sampling of parameter space; indeed, only a few parameter sets show substantial addressing power. However, with s=1 and therefore selection for parameter anticorrelations, the resulting histogram is notably shifted towards higher addressing power, despite starting from similar randomly chosen initial conditions. Longer runs of the evolutionary algorithm did not change the resulting histograms, indicating equilibration within 200 iterations.

### Quantification and statistical analysis

#### Flow cytometry data

Single-cell flow cytometry data were analyzed by taking the population median. For measured experimental responses (Figure 5), responses were quantified by taking the mean of at least 3 repeats. Fold change was measured compared to response with no ligand present and then normalized by the maximum fold change for each cell type.

#### Robustness

The robustness of top parameter sets for each bandwidth was analyzed by randomly perturbing receptor expression levels (STAR Methods: Analysis of robustness). ROC curves and AUC values are computed for 100 perturbations (Figures 3C and 3G), along with the corresponding distinguishability values (Figure S3B).

#### Parameter correlations

To analyze potential relationships between parameters, two correlation metrics were computed for each of 65,536 parameter sets (STAR Methods: Analysis of parameter correlations). To evaluate the association of these correlations with mutual information, parameter sets were sorted by mutual information and binned into sets of 800 (apart from the last bin, with a bin size of 736). These bins were then analyzed by plotting the mean correlation and the mean mutual information (Figures 6G and 6H).

## Data Availability

• All data have been deposited at the CaltechDATA research data repository (https://doi.org/10.22002/D1.1692) and are publicly available as of the date of publication. The DOI is listed in the key resources table. • All original code has been deposited at GitHub (https://github.com/christinasu/PromiSys) as well as the CaltechDATA research data repository (https://doi.org/10.22002/D1.20047) and is publicly available as of the date of publication. The DOI is listed in the key resources table. • Any additional information required to reanalyze the data reported in this paper is available from the lead contact upon request.

## Supplemental information

Document S1. Figures S1–S6 and Table S1

Document S2. Article plus supplemental information
