# Supplementary material for: Ligand-receptor promiscuity enables cellular addressing
Source: Cell Syst. 2022 May 18;13(5):408–425.e12. doi: 10.1016/j.cels.2022.03.001 (PMC10897978; doi:10.1016/j.cels.2022.03.001)
Supplement: Document S1. Figures S1–S6 and Table S1 [file mmc1.pdf]

Cell Systems, Volume *13*

Supplemental information

Ligand-receptor promiscuity enables cellular addressing

Christina J. Su, Arvind Murugan, James M. Linton, Akshay Yeluri, Justin Bois, Heidi Klumpe, Matthew A. Langley, Yaron E. Antebi, and Michael B. Elowitz

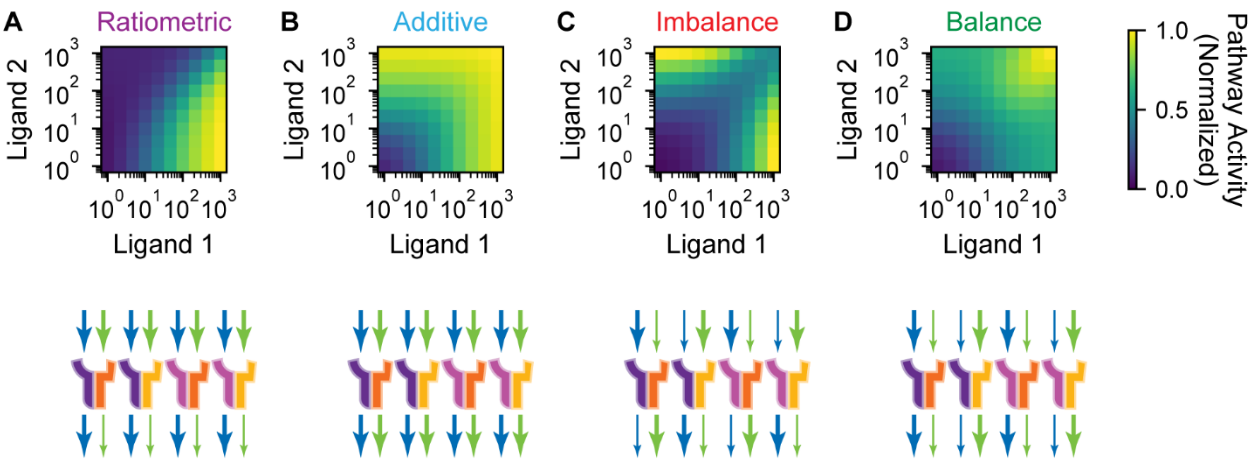

Figure S1: Promiscuous ligand-receptor interactions generate a repertoire of archetypal response functions. Related to Figure 2.

Four archetypal response functions—ratiometric, additive, imbalance, and balance—appeared in a more complex model of the BMP signaling pathway (Antebi et al., 2017). Here, we show that similar archetypes appear in the model used here (top) and illustrate the model parameters that generate them (bottom). Parameter diagrams represent the affinity (top arrows) and activity (bottom arrows) parameters corresponding to each signaling complex. Arrow width indicates relative magnitude. Thin arrows correspond to values of 0.1, while thick arrows represent values of 1.

(A) In ratiometric responses, one ligand reduces the activity of the other, such that the overall response approximates the ratio of the two concentrations. Such responses can arise through competitive inhibition, where a second ligand binds the receptors that are needed to generate signaling activity but produces inactive signaling complexes.

(B) Additive responses approximate the sum of the two ligand concentrations, as the ligands increase pathway activity either alone or together. Ligands that activate receptors equivalently can generate such responses.

(C) In imbalance detection, the pathway is most active when there is a large imbalance in the levels of the two ligands. These responses can arise if, for instance, competition between two ligands favors complexes with low signaling activity.

(D) Balance detection responses show most activity when both ligands are present simultaneously at a particular ratio. One mode for generating them is when ligand binding favors formation of high-activity signaling complexes.

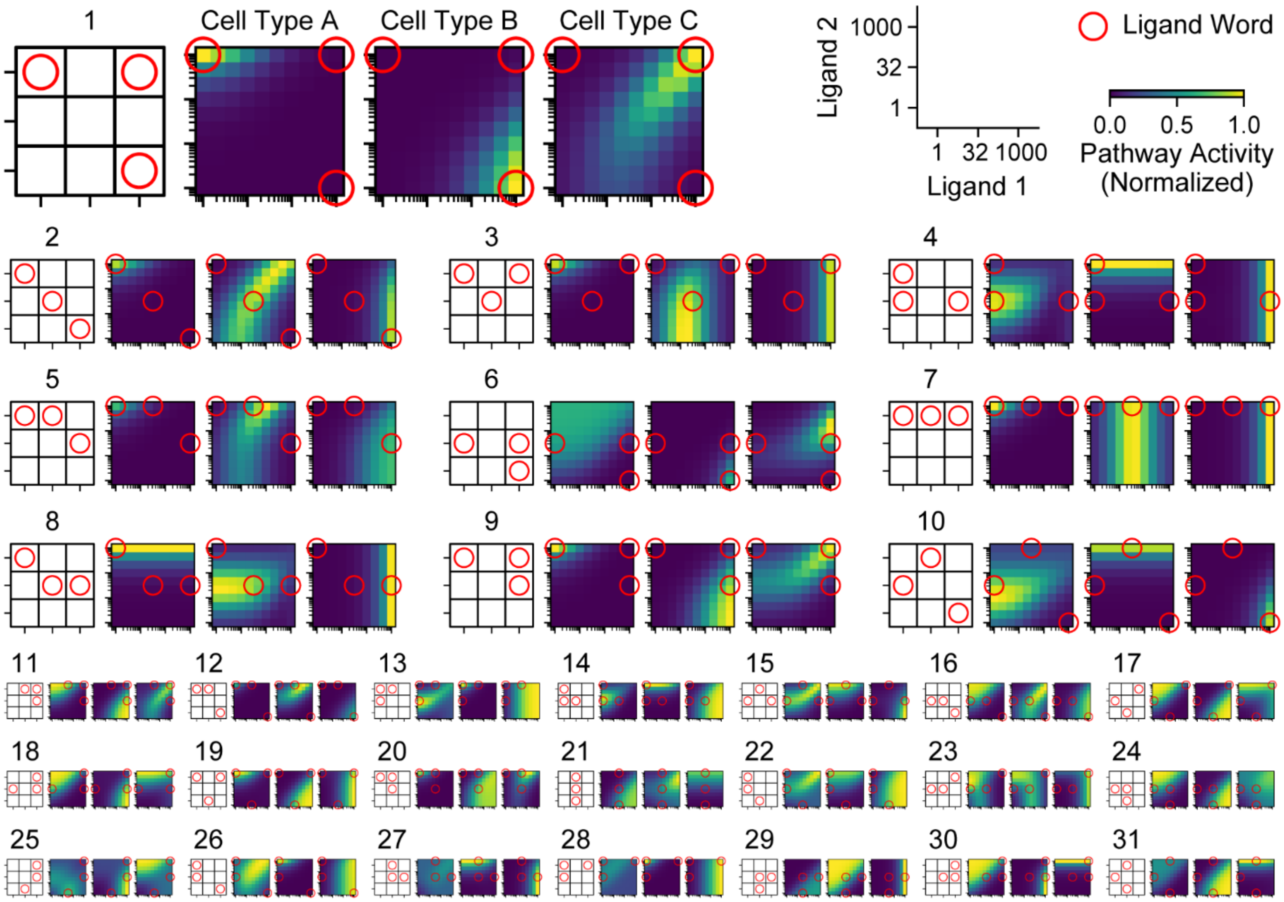

Figure S2: Orthogonal addressing can arise from a variety of different response types. Related to Figure 2.

For the parameter sets represented in Figure 2D, the responses of each cell type are shown. Parameter sets are ordered by distinguishability, from best to worst. These responses illustrate that three-channel addressing can be achieved in a variety of ways, although common patterns do emerge (for example, schemes 1-2 and schemes 3-4). Different scales are used to focus on the strongest examples.

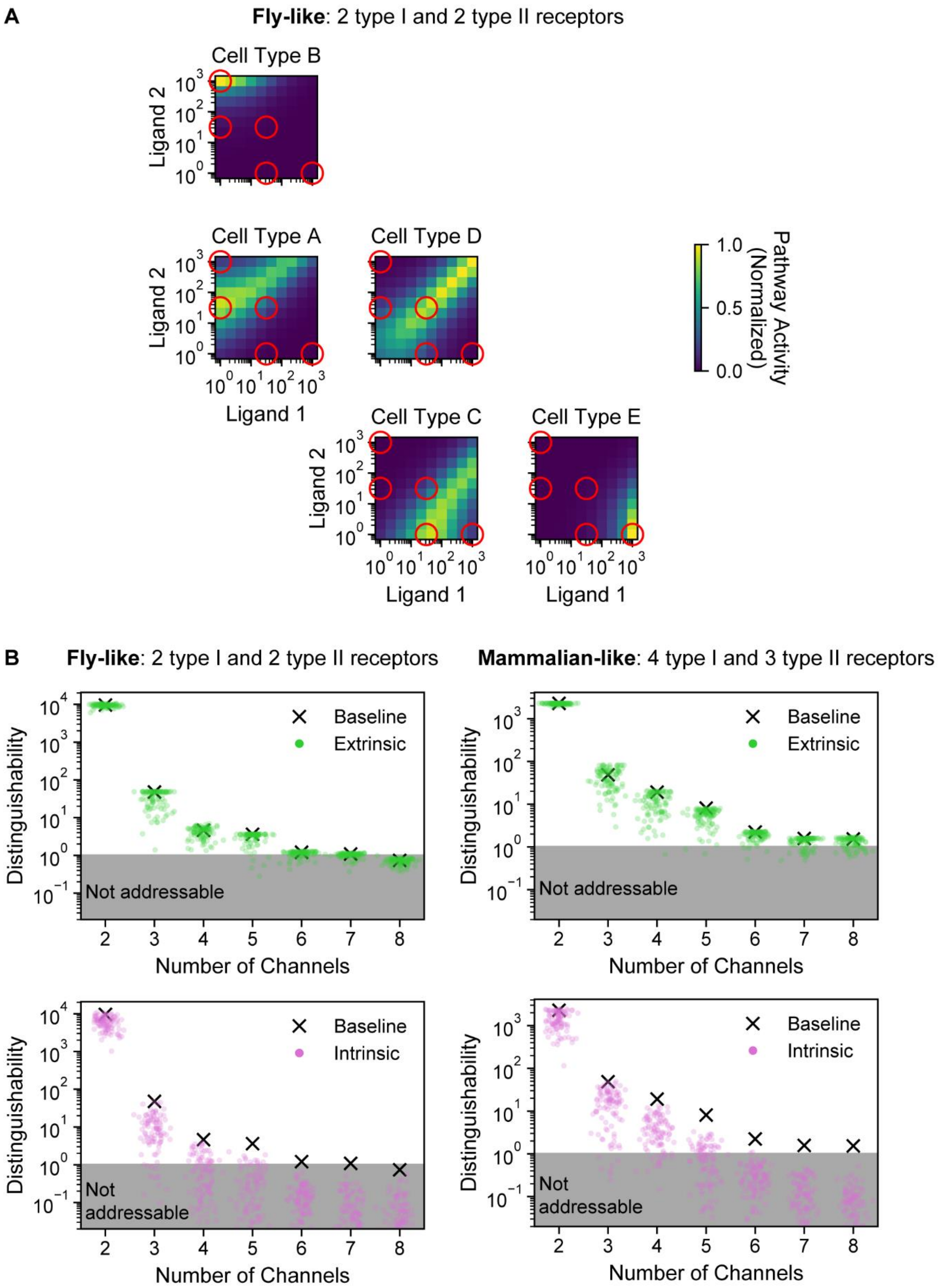
A
Fly-like: 2 type I and 2 type II receptors
Cell Type B
Cell Type A
Cell Type D
Cell Type C
Cell Type E
Ligand 1
Ligand 2
Pathway Activity (Normalized)
B
Fly-like: 2 type I and 2 type II receptors
Mammalian-like: 4 type I and 3 type II receptors
Baseline
Extrinsic
Intrinsic
Distinguishability
Not addressable
Number of Channels

Figure S3: Orthogonal addressing schemes are robust to extrinsic noise in receptor expression levels. Related to Figure 3.

(A) The responses of each cell type in the five-channel system analyzed in Figure 3D are shown. As in Figure 3I, ligand words corresponding to orthogonally activating channels are shown as red circles. Responses have been rearranged such that the response of a given cell type is shown in the relative position of its orthogonally activating ligand word. For example, the top left cell type is activated by high levels of ligand 2 only, while the bottom right cell type is orthogonally activated by high levels of ligand 1 only.

(B) Top parameter sets from (left) the fly-like model (two type I and two type II receptor variants) of Figures 3A-C and (right) the mammalian-like system (four type I and three type II receptor variants) of Figures 3E-G were evaluated for addressing specificity in the presence of noise. Receptor expression levels were perturbed with correlated extrinsic (top, green) or uncorrelated intrinsic (bottom, purple) noise (STAR Methods: Analysis of Robustness), and distinguishability values were computed with all other parameters held constant. For each condition, the results of 100 perturbations are shown, along with the baseline value (black crosses).

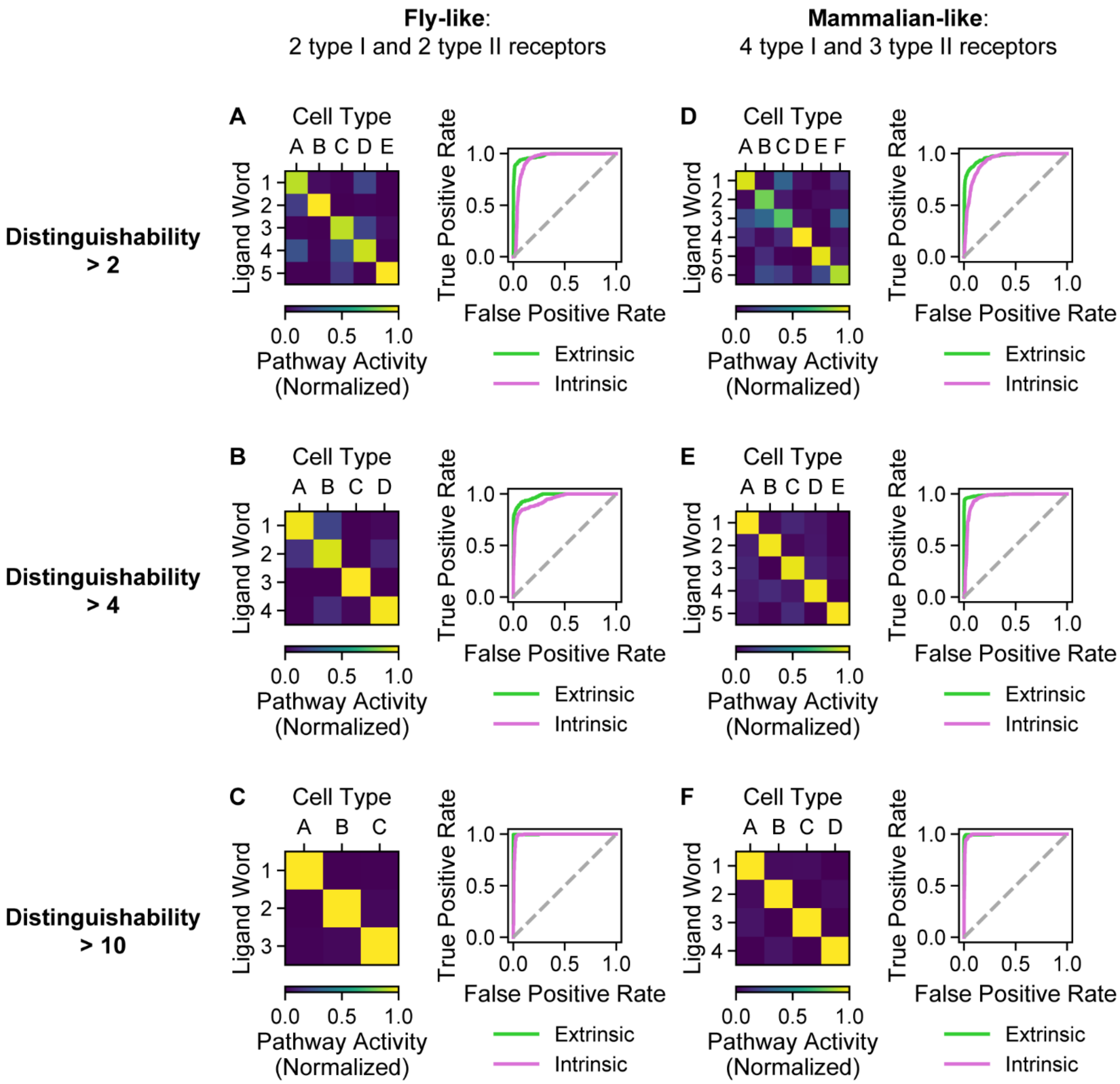

Figure S4: Varying distinguishability thresholds reveal a tradeoff between the capacity and robustness of addressing systems. Related to Figure 3.

To understand the tradeoff between addressing capacity and robustness, we show the system of highest bandwidth that exceeds the indicated distinguishability thresholds. Specifically, we highlight the crosstalk matrix as well as the ROC curves in response to extrinsic (green) or intrinsic (purple) noise.

(A) In the fly-like model, five channels can be orthogonally addressed at a distinguishability of at least 2.

(B) In the fly-like model, four channels can be orthogonally addressed at a distinguishability of at least 4.

(C) In the fly-like model, three channels can be orthogonally addressed at a distinguishability of at least 10.

(D) In the mammalian-like model, six channels can be orthogonally addressed at a distinguishability of at least 2.

(E) In the mammalian-like model, five channels can be orthogonally addressed at a distinguishability of at least 4.

(F) In the mammalian-like model, four channels can be orthogonally addressed at a distinguishability of at least 10.

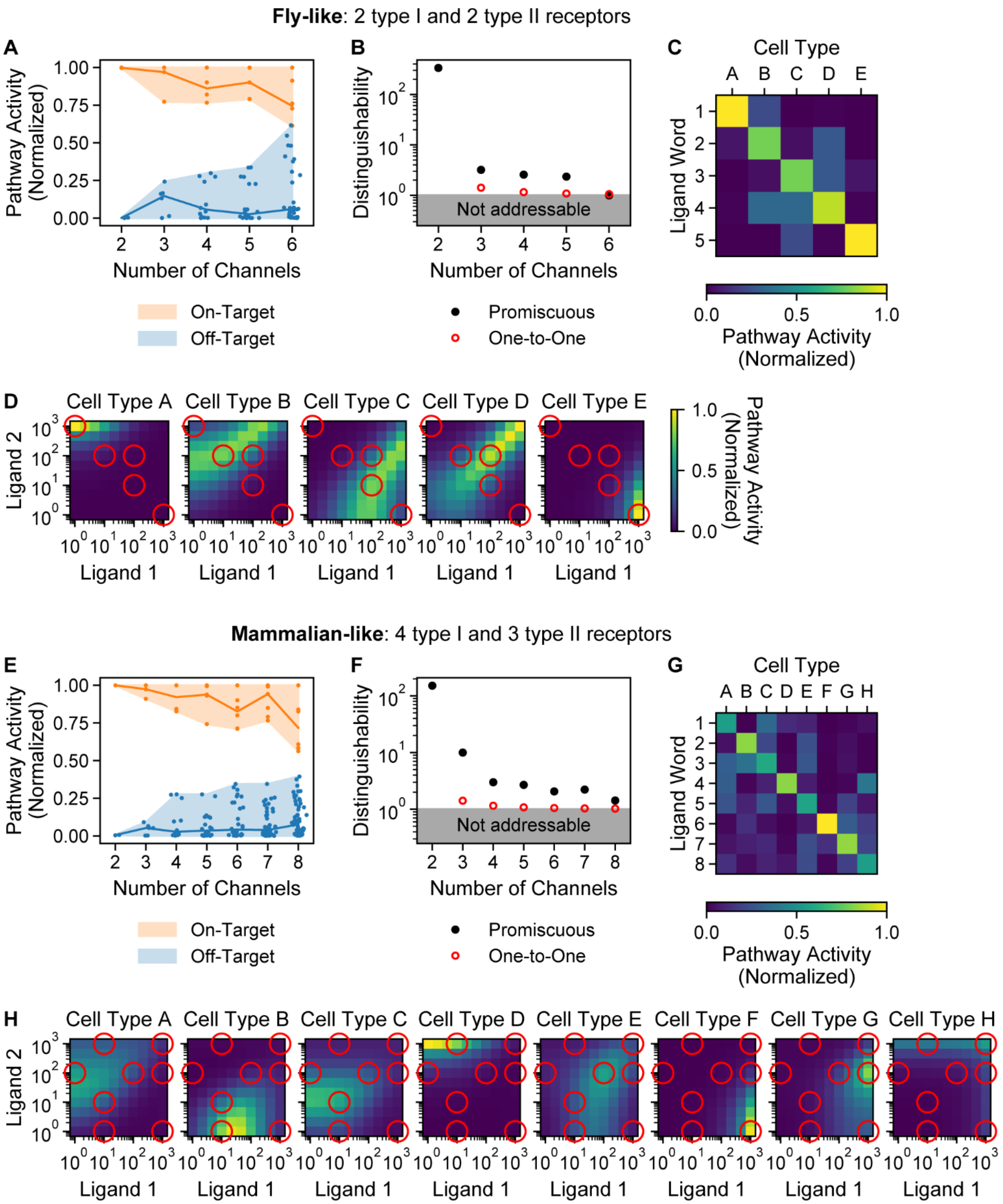
Fly-like: 2 type I and 2 type II receptors
A
Pathway Activity (Normalized)
Number of Channels
On-Target
Off-Target
B
Distinguishability
Not addressable
Number of Channels
Promiscuous
One-to-One
C
Cell Type
A B C D E
Ligand Word
Pathway Activity (Normalized)
D
Cell Type A
Cell Type B
Cell Type C
Cell Type D
Cell Type E
Ligand 2
Ligand 1
Pathway Activity (Normalized)
Mammalian-like: 4 type I and 3 type II receptors
E
Pathway Activity (Normalized)
Number of Channels
On-Target
Off-Target
F
Distinguishability
Not addressable
Number of Channels
Promiscuous
One-to-One
G
Cell Type
A B C D E F G H
Ligand Word
Pathway Activity (Normalized)
H
Cell Type A
Cell Type B
Cell Type C
Cell Type D
Cell Type E
Cell Type F
Cell Type G
Cell Type H
Ligand 2
Ligand 1

Figure S5: Orthogonal addressing capacity remains generally consistent when considering higher-resolution ligand discretization. Related to Figure 3.

(A) We sought to optimize for orthogonal addressing systems using a four-level ligand grid, compared to the three concentration levels used throughout the rest of this work. We optimized randomly generated addressing schemes for each given number of channels, moving to a higher bandwidth once a parameter set had been successfully optimized. On-target and off-target responses are shown for the best parameter set for each number of channels in a fly-like model, as in Figure 3A.

(B) Distinguishability values are plotted for each number of channels, as in Figure 3B. The five-channel system, which reflects the highest addressable bandwidth and has a distinguishability of 2.3, is further analyzed in (C-D).

(C) The crosstalk matrix is shown for the five-channel system, as in Figure 3D.

(D) The full responses of each cell type are shown for the five-channel system. Red circles correspond to the five ligand words.

(E) The pathway activities for the top parameter set of each tested bandwidth are shown for a mammalian-like model with four type I and three type II receptors, as in (A).

(F) Distinguishability values are plotted for the mammalian-like model, as in (B). The eight-channel system, which reflects the highest addressable bandwidth and has a distinguishability of 1.4, is further analyzed in (G-H).

(G) The crosstalk matrix for the example in the mammalian-like model is shown, as in (C).

(H) The full responses for the example in the mammalian-like model are shown, as in (D).

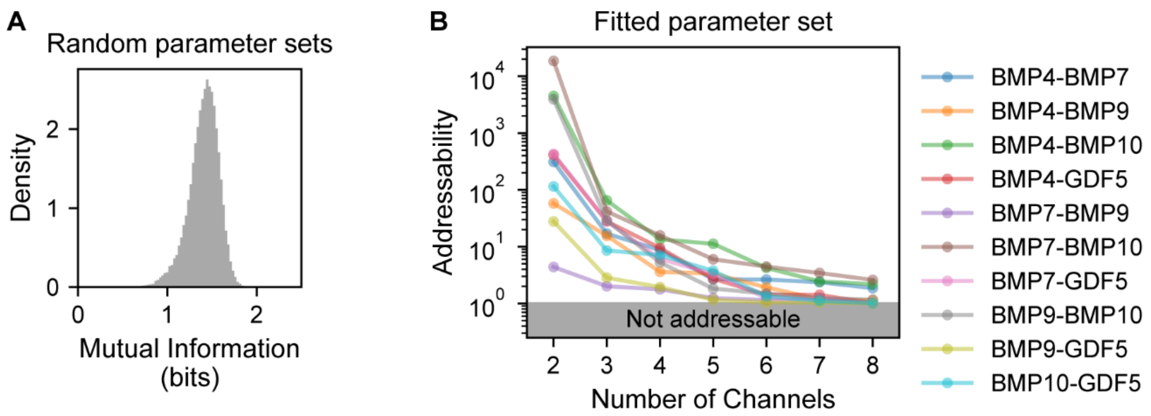

Figure S6: Addressing properties vary across parameter sets. Related to Figure 6.

(A) The approach of Figures 6A-B was applied to an equivalent number of randomly generated parameter sets, rather than a grid of parameter values. The resulting distribution of mutual information values is similar, indicating that the result is robust to the method of parameter sampling (cf. Figure 6B).

(B) Parameters for five ligands (indicated in legend), two type I receptors, and three type II receptors were fitted to the experimental measurements of BMP responses in multiple cell lines with differing receptor expression profiles described in (Klumpe et al., 2022) and analyzed for their addressing potential. Specifically, we computed the addressability for every pair of ligands using the same libraries of ligand combinations and cell types as in (A). Different pairs show varying levels of addressing potential, with some pairs (e.g., BMP4-BMP7, BMP4-BMP10, and BMP7-BMP10) exhibiting addressing of different cell type groups for every ligand combination and others (e.g., BMP7-BMP9) showing lower bandwidths.

Table S1: Experimentally analyzed cell lines have receptor expression profiles resembling those of biological cell types. Related to Figure 5.

Cell lines analyzed in Figure 5 were compared to cell types from annotated single-cell RNA-seq expression atlases (Tabula Muris Consortium, 2020; Tabula Muris Consortium et al., 2018). Receptor profiles for NMuMG cells were measured directly through bulk RNA-seq, while receptor profiles for perturbed NMuMG lines were simulated by estimating the perturbation magnitudes from qPCR data (Klumpe et al., 2022). For each cell line, a cell type with similar receptor expression profile is identified, along with the relevant developmental timepoint or age.

**Cell Line**	**Similar Cell Type**
NMuMG	Tongue keratinocyte (24 months)
ACVR1 KD	Tongue basal cell of epidermis (3 months)
BMPR2 KD	Tongue keratinocyte (18 months)
ACVRL1 OX	Limb muscle Schwann cell (18 months)
